# Inhibition of GSK3 Represses the Expression of Retinoic Acid Synthetic Enzyme ALDH1A2 via Wnt/β-Catenin Signaling in WiT49 Cells

**DOI:** 10.3389/fcell.2020.00094

**Published:** 2020-03-17

**Authors:** Yifan Li, Hui Gong, Jiangfeng Ding, Fujuan Zhao, Jihui Du, Jun Wan, Juan Zhang, Shaoxiong Liu, Jing Li, Lei Wang, Bei Zhou

**Affiliations:** ^1^Central Laboratory, Huazhong University of Science and Technology Union Shenzhen Hospital and the Affiliated Shenzhen Sixth Hospital of Guangdong Medical University, Shenzhen, China; ^2^Shenzhen Key Lab of Endogenous Infection, Huazhong University of Science and Technology Union Shenzhen Hospital and the Affiliated Shenzhen Sixth Hospital of Guangdong Medical University, Shenzhen, China; ^3^Department of Stomotology, Huazhong University of Science and Technology Union Shenzhen Hospital and the Affiliated Shenzhen Sixth Hospital of Guangdong Medical University, Shenzhen, China; ^4^Department of Pathology, Huazhong University of Science and Technology Union Shenzhen Hospital and the Affiliated Shenzhen Sixth Hospital of Guangdong Medical University, Shenzhen, China; ^5^Shenzhen Key Laboratory for Neuronal Structural Biology, Biomedical Research Institute, Shenzhen Peking University–The Hong Kong University of Science and Technology Medical Center, Shenzhen, China; ^6^Division of Life Science, The Hong Kong University of Science and Technology, Kowloon, Hong Kong; ^7^Department of Endocrinology, Huazhong University of Science and Technology Union Shenzhen Hospital and the Affiliated Shenzhen Sixth Hospital of Guangdong Medical University, Shenzhen, China

**Keywords:** ALDH1A2, RALDH2, fetal kidney, Wnt signaling, β-catenin, GSK3 inhibitor, retinoic acid

## Abstract

Organogenesis, including renal development, requires an appropriate retinoic acid concentration, which is established by differential expression of aldehyde dehydrogenase 1 family member A2 (*ALDH1A2*) and cytochrome P450 family 26 subfamily A/B/C member 1 (*CYP26A1/B1/C1*). In the fetal kidney, *ALDH1A2* expresses in the developing stroma and renal vesicle and its derivatives but does not present in the ureteric bud. It remains unclear what may contribute to this expression pattern. Here we show that the glycogen synthase kinase 3 alpha/beta (GSK3A/B) inhibitor CHIR99021 significantly represses *ALDH1A2* expression in WiT49, which is a Wilms’ tumor cell line that exhibits “triphasic” differential potential and is used as a fetal kidney cell model. CHIR99021 fails to suppress *ALDH1A2* as β-catenin is inhibited, suggesting that the downregulation of ALDH1A2 by CHIR99021 is through Wnt/β-catenin signaling. Ectopic expression of mouse Wnt1, Wnt3a, Wnt4, and Wnt9b represses *ALDH1A2* expression in WiT49 cells. Using immunohistochemistry, we show an inverse correlation of *Aldh1a2* expression with β-catenin in rat E18.5 kidney. ChIP demonstrated that β-catenin is recruited to the *ALDH1A2* promoter, the conserved intron1G, and another site within intron 1 of *ALDH1A2*. Using a luciferase assay, we further show that the *ALDH1A2* promoter and the intron1G element are involved in the repression of *ALDH1A2* expression by CHIR99021. Our work demonstrates that *ALDH1A2* expression can be directly repressed by the Wnt/β-catenin signaling in fetal kidney cells, suggesting that Wnt/β-catenin may play a role in maintaining the expression pattern of *ALDH1A2* in the fetal kidney, thus controlling the availability and localization of retinoic acid and regulating aspects of kidney development.

## Introduction

Renal development is initiated by mutual signaling interactions between the ureteric bud derived from the Wolffian duct and its surrounding mesenchyme. The Wolffian duct extends caudally and interacts with its adjacent mesenchyme to generate a pronephric, mesonephric, and, finally, the metanephric kidney. The metanephros grows and differentiates to make up the functional kidney in adult. Metanephric mesenchyme differentiates and becomes the condensed mesenchyme (cap mesenchyme), which forms lineages of epithelial, stromal, and endothelial cells. The epithelial cell lineage differentiates into structures that include the renal vesicle, pretubular aggregate, comma-shaped and S-shaped bodies, and renal nephron, including podocyte on the renal vesicle layer of the glomerulus, proximal convoluted tubule, loop of Henle, and distal convoluted tubule. Ureteric buds develop to form collecting duct, renal calyces, pelvis, and ureter (Davidson, 2008; Dressler, 2009). Kidney development is a continuous process in the outer cortex of the developing kidney, where signaling interaction among cell types, including mesenchyme, ureteric bud, and stroma, continues until birth in human or after birth in mouse (Cullen-Mcewen et al., 2016).

Renal development depends on all-trans retinoic acid (atRA) signaling. atRA mainly acts as a ligand for nuclear retinoic acid receptors (RARs), which consists of RARα, RARβ, and RARγ, resulting in a change in subsequent biological procedures and gene transcription (Dolle, 2009). atRA can also act through non-canonical pathways including binding to the PPARδ/β (peroxisome proliferator-activated receptor delta/beta) receptor in the nucleus (Napoli, 2017) or binding to the RARα/Gαq (G protein alpha Q) complex on the cell membrane lipid raft to activate the p38MAPK (Piskunov and Rochette-Egly, 2011) and the PI3K pathway (Masiá et al., 2007). The knockout of both *Rara* and *Rarb* results in the hypoplasia/agenesis of the kidney (Mendelsohn et al., 1999). The impact of retinoic acid in renal development is known to mediate through the upregulation of ret proto-oncogene (*Ret*) expression on the cell membrane of the ureter bud. Ret is a receptor that binds its ligand, glial cell line-derived neurotrophic factor (Gdnf), secreted from the cap mesenchyme (Batourina et al., 2001; Rosselot et al., 2010) and stromal cells (Magella et al., 2018). Endogenous atRA is produced by two stages of oxidation of vitamin A (all-trans-retinol) via an intermediate state, all-trans retinal (Duester, 2008; Napoli, 2012). The second stop of retinoic acid synthesis is catalyzed by enzymes including aldehyde dehydrogenase 1 family member A1 (ALDH1A1) (Fan et al., 2003), aldehyde dehydrogenase 1 family member A2 (ALDH1A2) (Wang et al., 1996; Zhao et al., 1996), and aldehyde dehydrogenase 1 family member A3 (ALDH1A3) (Grun et al., 2000; Sima et al., 2009). *Aldh1a2*^–/–^ mutant mouse kidney exhibits a significant reduction of ureteric buds and nephrons (Rosselot et al., 2010). *Aldh1a3*^–/–^ has no or little effect on renal development (Rosselot et al., 2010). *Aldh1a1*^–/–^ mutant is non-lethal, adults are fertile, and no abnormality has been reported (Fan et al., 2003; Matt et al., 2005). These data suggest that Aldh1a2 activity is the primary source of retinoic acid during kidney development (Rosselot et al., 2010). The retinoic acid can be inactivated by cytochrome P450 family 26 (CYP26) subfamily A/B/C member 1 (CYP26A1/B1/C1). Excessive retinoic acid is teratogenic (Piersma et al., 2017) and causes hypoplastic and polycystic kidney (Fantel et al., 1977; Lee et al., 2012). Therefore, the deficiency or excess of retinoic acid signaling leads to abnormalities in kidney development, suggesting that the proper regulation of retinoic acid concentration and retinoic acid synthetic enzymes is critical.

Regulation of *Aldh1a2* expression is important for pronephros development. In zebrafish pronephros, *cdx* mutation leads to *Aldh1a2* upregulation and disrupts pronephros positioning and distal segment formation (Wingert et al., 2007), suggesting that inhibition of *Aldh1a2* expression by cdx is critical for pronephros development. Recently, it was shown that anterior kidney fates are induced by retinoic acid on the dorsal side of the embryo with *Aldh1a2* expression, while posterior kidney progenitors are protected by the Cyp26a1 enzyme (Naylor et al., 2016). These studies suggest that *Aldh1a2* expression determines the precise localization of retinoic acid signaling and that the repression of the *Aldh1a2* is necessary for the pronephros formation of zebrafish. However, studies of the regulation of *Aldh1a2* expression within the metanephric kidney remains rare.

In the metanephric kidney, as well as being strongly expressed in the stromal cells of the outer cortex, *Aldh1a2* is weakly presented in the proximal section of the comma-shaped body, vigorously but transiently expressed in glomerular anlagen in the S-shaped body and the visceral layer of the glomerulus in stage III nephron, and declines aggressively in the podocyte of stage IV nephron. Its expression in collecting tubules during E18-P4 has also been observed (Batourina et al., 2001; Niederreither et al., 2002; Marlier and Gilbert, 2004; Rosselot et al., 2010). This intricate expression pattern of *Aldh1a2* should be tightly controlled. It was reported that *Foxd1* knockout led to the repression of *Aldh1a2* expression in stromal cells of the fetal kidney (Levinson et al., 2005; Fetting et al., 2013). Recently we provide *in vitro* evidence that ectopic expression of WT1 transcription factor (*WT1*) represses *ALDH1A2* expression in the human fetal kidney cell line HEK293 (Li et al., 2017). It is important to note that, as the retinoic acid concentration is dependent on the expression of enzymes like ALDH1A2, repression of *ALDH1A2* is as vital as its upregulation for the formation of the correct local concentration of retinoic acid in kidney development. The regulator responsible for the lack of *ALDH1A2* expression in ureteric bud and cap mesenchyme remains unclear.

Wnt signaling is essential for fetal kidney development (Halt and Vainio, 2014). The canonical Wnt/β-catenin pathway is the most well-characterized Wnt pathway (Nusse and Clevers, 2017). The signal is executed by translocation of β-catenin from plasma into the nucleus and acts together with TCF or other unknown factors to regulate gene expression. Observations show that *Wnt-2b, -4, -5a, -6, -7b, -9b*, and *-11* are expressed during kidney development (Kispert et al., 1996; Kispert et al., 1998; Lin et al., 2001; Itaranta et al., 2002; Carroll et al., 2005). Wnt1, -3a, -4, -7a, and -7b were reported to trigger nephrogenesis in the metanephric mesenchyme (Kispert et al., 1998). Wnt4 was secreted by metanephric mesenchyme. The loss of *Wnt4* leads to the failure of the formation of pretubular cell aggregates (Stark et al., 1994). *Wnt9b* is expressed in ureteric buds and activates the canonical Wnt signaling pathway in the surrounding metanephric mesenchyme (Kispert et al., 1998; Carroll et al., 2005; Park et al., 2007). Loss of *Wnt9a* leads to failure of nephron formation. It also plays an important role in the convergent extension of renal tubular epithelial cells. Wnt6, Wnt7b, and Wnt11 are generated by ureteric bud cells. Wnt2b is secreted by developing stromal cells (Halt and Vainio, 2014).

Recently, increasing evidence suggests that the canonical Wnt signaling may regulate *Aldh1a2* expression. It was shown that, in 293T cells, β-catenin and TCF4 activate mouse *Aldh1a2* promoter (Hong et al., 2015). In the Six2 positive pre-tubular aggregate of the embryonic kidney, β-catenin/LEF/TCF complex and Six2 were recruited to a region within the first intron of the *Aldh1a2* gene (chr9:71065207-71065805) (Park et al., 2012). This region partially overlapped with a TCF7L2 (TCF4) bound region identified in HEK293 cells using ChIP-sequencing (Frietze et al., 2012). It was previously found that in the first intron of the *Aldh1a2* gene, there is an enhancer termed intron1G that can activate *Aldh1a2* expression in the mouse developing neuron cells. Intron1G contains at least three predicted LEF/TCF binding motif (Castillo et al., 2010).

As Wnt signaling and retinoic acid signaling are critically involved in kidney development and disease and the above data suggest that the canonical Wnt signaling may regulate *Aldh1a2*, we hypothesized that Wnt/β-catenin signaling might regulate *Aldh1a2* expression in the developing kidney. In this study, we focus on examining whether the canonical Wnt signaling can control *Aldh1a2* expression in the fetal kidney context. We report for the first time that *Aldh1a2* expression can be suppressed by CHIR99021 via the canonical Wnt signaling. We confirm that *Aldh1a2* repression by Wnt signaling involves the *Aldh1a2* promoter and intron1G element. Our data indicate that Wnt signaling could be upstream of retinoic acid signaling during kidney development. Our findings have implications for linking the Wnt and retinoic acid pathways during kidney development.

## Materials and Methods

### Chemicals

The GSK3 inhibitor CHIR99021 (Cat. SML1046, Sigma-Aldrich Corp., St. Louis, MO, United States) was obtained from Sigma. CHIR99021 was dissolved in dimethyl sulfoxide (DMSO) (Cat.196055, MP Biomedicals). CHIR99021 is an aminopyrimidine derivative that is a highly potent inhibitor of GSK3. It inhibits GSK3β (IC_50_ = 6.7 nM) and GSK3α (IC_50_ = 10 nM), acting as a Wnt activator.

### Primers

The primers for real-time PCR used in this study are listed in Table 1.

**TABLE 1:** Primers for real-time PCR.

**Name of primer**	**Primer sequence (5′ to 3′)**
ALDH1A2-QF2	GGGCAGTTCTTGCAACCATGGAAT
ALDH1A2-QR2	TTTGATGACGCCCTGCAAATCCAC
TBPRQF	GCCCGAAACGCCGAATAT
TBPRQR	CCGTGGTTCGTGGCTCTCT
CTNNB1-QF1	CTTCACCTGACAGATCCAAGTC
CTNNB1-QR1	CCTTCCATCCCTTCCTGTTTAG
ALDH1A2_CHIP_QF1	GGGACGATAGCTCTTAGCGTGTAA
ALDH1A2_CHIP_QR1	TGCTTCGTGGGCTCCTTTAGTTCT
MYC-ChIP-QF2	CCCGTCTAGCACCTTTGATTT
MYC-ChIP-QR2	ACACGGAGTTCCCAATTTCTC
1A2-in1G-ChIPQF1	CTCCCAAGCTGATTTATGGTCT
1A2-in1G-ChIPQR1	AGGCCATTCCCATTTACTATCC
1A2-in1G-ChIPQF2	TCTCTGGGTGTTGTCCAATTT
1A2-in1G-ChIPQR2	CCGTCCTAAACACCAGATGAA
1A2-in1G-ChIPQF3	TTTAGAGCTAGGCCACAGAATG
1A2-in1G-ChIPQR3	AGCGATGACACATAGCAAGAA
1A2-in1G-ChIPQF4	GGGATTCCAGGTTCTTGCTATG
1A2-in1G-ChIPQR4	CAGACTCCAATGGCTGTTGATTA
1A2-in1-six2bcat-ChIPQF1	TTTAGGAACGAGCAGGGAAAG
1A2-in1-six2bcat-ChIPQR1	CCTTCCCAAATCTGAGGGTTAG
1A2-in1-six2bcat-ChIPQF2	CCTAACCCTCAGATTTGGGAAG
1A2-in1-six2bcat-ChIPQR2	AGGGAGTAAGAGAACTGGAAGA
1A2-in1-six2bcat-ChIPQF3	CCCAGTAGGAAAGACAACTGAT
1A2-in1-six2bcat-ChIPQR3	GTGGGAAAGTGGATACTGGATAA
1A2-in1-six2bcat-ChIPQF4	CCTTGACAAACAACCCTCATAAA
1A2-in1-six2bcat-ChIPQR4	GCTGGGAATGCTCACAGATA

### Plasmids

M50 Super 8x TOPFlash (Addgene #12456) and M51 Super 8x FOPFlash (TOPFlash mutant) (Addgene #12457) were gifts from Randall Moon (Veeman et al., 2003). The HSV-thymidine kinase promoter (pRL-TK) (Promega), kindly provided by Dr. Kun Du, was used as a Renilla luciferase control reporter.

Expression plasmids of multiple Wnts, including pRK5-mWnt1 (Addgene #42273), pRK5-mWnt2b (Addgene #42275), pRK5-mWnt3a (Addgene #42277), pRK5-mWnt4 (Addgene #42278), pRK5-mWnt5a (Addgene #42279), pRK5-mWnt6 (Addgene #42281), pRK5-mWnt7a (Addgene #42282), pRK5-mWnt7b (Addgene #42283), pRK5-mWnt9b (Addgene #42287) and pRK5-mWnt11 (Addgene #42290), were gifts from Chris Garcia and Jeremy Nathans (Yu et al., 2012). pRK5 empty vector was made by re-ligation of the backbone of pRK5-mWnt4 (Addgene #42278) digested by *Pst*I.

*ALDH1A2* promoter or intron1G was cloned using the primers shown in Table 2. Promoter construct pGL3B-1A2ps (889 bp) was made by digestion of pGL3B-1A2p with *Kpn*I/*Avr*II, followed by ligation of the promoter sequence to the pGL3-basic vector, which was digested with *Kpn*I/*Nhe*I. The MF2 and PF2 reporter vectors were made by joining the minus strand or the plus strand of *ALDH1A2* intron1G with the *ALDH1A2* promoter F2 using the overlap extension polymerase chain reaction method (OE-PCR) (Figure 6A) followed by ligation with pGL3-basic plasmid using *Kpn*I/*Hin*dIII enzyme sites.

**TABLE 2:** Primers for *ALDH1A2* promoter and intron1G cloning.

**Name of promoter**	**Name of primer**	**Primer sequence (5′ to 3′)**	**Product length (bp)**
F1	*Kpn*I-1A2-Gaudix-F1	aaaaggtaccATGTTTTCTGGCTGATGCTTAATGT	1623
	*Xho*I-1A2-Gaudix-R1	aaaactcgagATCTTGCTGGAAGTCATGGTG	
F2	*Kpn*I-1A2-Gaudix-F2	aaaaggtaccTAGGCCCCTGTCAAGCTTACATT	1321
	*Xho*I-1A2-Gaudix-R1	aaaactcgagATCTTGCTGGAAGTCATGGTG	
QF1	*Kpn*I-1A2-CHIPQF1	aaaaggtaccGGGACGATAGCTCTTAGCGT	1066
	*Xho*I-1A2-Gaudix-R1	aaaactcgagATCTTGCTGGAAGTCATGGTG	
1A2p	*Kpn*I-1A2_CHIP_QF1	aaaaggtaccGGGACGATAGCTCTTAGCGT	1156
	*Hin*dIII-1A2_CHIP_QR4	ttttaagcTTAATTTCGAGATTGGGCGTGG	
P	*Kpn*I-1A2-intron1G-plusF	aaaaggtaccTGTCTCTTGACATCTGTTTTAGGAA	1286
	*Hin*dIII-1A2-intron1G-plusR	ttttaagcttCTTCCTCTCCTTTTGCATGTGACT	
M	*Kpn*I-1A2-intron1G-plusR	aaaaggtaccCTTCCTCTCCTTTTGCATGTGACT	1286
	*Hin*dIII-1A2-intron1G-plusF	ttttaagcttTGTCTCTTGACATCTGTTTTAGGAA	
PF2	*Kpn*I-1A2p-intron1G-plusF	aaaaggtaccTGTCTCTTGACATCTGTTTTAGGAA	1286
	1A2-intron1G-plusR	CTTCCTCTCCTTTTGCATGTGACT	
	1A2-In1GpR-GaudixF2	AGTCACATGCAAAAGGAGAGGAAGTAGGCCCCTGTCAAGCTTACATT	1321
	*Xho*I-1A2-GaudixR1	aaaactcgagATCTTGCTGGAAGTCATGGTG	
MF2	*Kpn*I-1A2p-intron1G-plusR	aaaaggtaccCTTCCTCTCCTTTTGCATGTGACT	1286
	1A2-intron1G-plusF	TGTCTCTTGACATCTGTTTTAGGAA	
	1A2-In1GpF-GaudixF2	TTCCTAAAACAGATGTCAAGAGACATAGGCCCCTGTCAAGCTTACATT	1321
	*Xho*I-1A2-GaudixR1	aaaactcgagATCTTGCTGGAAGTCATGGTG	

*Underlined sequences are restriction enzyme sites.*

### siRNAs

siRNAs targeting human *CTNNB1* exons were designed and synthesized by GenePharma (Shanghai, China). The siRNA oligos include *CTNNB1* siRNA-1 5′-GGA CAC AGC AGC AAU UUG UTT-3′, *CTNNB1* siRNA-2 5′-GCU GCU UUA UUC UCC CAU UTT-3′, and the negative control siRNA oligo 5′-UUC UCC GAA CGU GUC ACG UTT-3′. The final concentration of siRNA for transfection is 50 nmol/L. Lipofectamine^®^ RNAiMAX reagent (Invitrogen) was used for siRNA transfection according to the manufacturer’s instruction.

### Cell Culture and Transfection

The WiT49 and 293 cells were cultured as described previously (Li et al., 2017). The WiT49 cell line was donated by Dr. Herman Yeger. It is a Wilms’ tumor (WT) cell line that is derived from the first-generation xenograft of a human WT lung metastasis. Some differentiation potential is retained by WiT49 cells, displaying the so-called “triphasic” histology (epithelioid, stromal-like, and mesenchymal) when grown in tissue culture plates (Alami et al., 2003). It is also triphasic (Mengelbier et al., 2014) or biphasic with stromal and epithelial components dominant (Li et al., 2010) in orthotopic xenograft models, suggesting that WiT49 may mimic certain aspects of the developing kidney (Li et al., 2002; Rivera and Haber, 2005).

### Animals

The pregnant Sprague-Dawley rats (SD rats) were obtained from Shenzhen Peking University–The Hong Kong University of Science and Technology Medical Center. Animal welfare and experimental procedures were carried out following the Guide for the Care and Use of Laboratory Animals (Ministry of Science and Technology of China, 2006) and were approved by the animal ethics committee of Nanshan People’s Hospital.

### Real-Time RT-PCR

RNA was purified using TRIZOL reagent (Invitrogen), and its concentration and purity were assessed at 260/280 nm by applying Nanodrop (Thermo). A sample of 1 μg total RNA was reverse-transcribed with oligo (dT)_20_ at 50°C for 1 h using the ThermoScript RT-PCR system (Invitrogen). Comparative quantitative real-time PCR was carried out employing SYBR Green qPCR supermix universal (Invitrogen) using the ABI7500 real-time PCR system. The reaction mixture was comprised of 10 μl SYBR (Invitrogen), 50 nmol/l ROX reference dye, 0.2 μmol/l forward primer, 0.2 μmol/l reverse primer, and 5 μl of 1:10 diluted cDNA template. PCR cycling was as follows: 50°C for 2 min, a denaturing step of 95°C for 10 min, followed by 40 cycles of 95°C for 15 s, 58°C for 30 s, and 72°C for 30 s. Gene expression was quantified by applying the comparative Ct method, normalizing to the housekeeping gene TATA box binding protein (TBP). Assays were accomplished in triplicate.

### Western Blot

Western blot was performed using polyclonal antibodies against β-catenin (Cat. 51067-2-AP, Proteintech), ALDH1A2 (Cat. ab75674, Abcam), and GAPDH (Goodhere, China). Secondary antibodies, including anti-rabbit IgG (whole molecule) – anti-mouse IgG and peroxidase antibody (whole molecule) – peroxidase antibody, were obtained from Sigma. In brief, 1 × 10^6^ cells were lysed in 150 μl 1 × Cell Lysis Buffer (Cell Signaling Technology, Inc.) plus EDTA-free protease inhibitor cocktail (Roche). Protein concentrations were measured by employing the DC Protein Assay Kit (Bio-Rad). For each lane, 50 μg proteins were denatured in 30 μl sample buffer (60 mmol/l Tris pH 6.8, 10% glycerol, 2% SDS, 5% mercaptoethanol), followed by incubation at 100°C for 5 min, and cooling on ice to denature proteins. Protein samples were loaded for electrophoresis and transferred to Immobilon-P Transfer Membrane (Millipore) with a wet transfer apparatus (Bio-Rad). The Immobilon-P membrane was blocked with 1% bovine serum albumin (BSA) (Sigma) in phosphate-buffered saline (PBS) for 2 h at room temperature. The membrane was then probed with primary antibodies overnight at 4°C at a dilution of 1:1000 or 1:200, followed by incubation with secondary antibody at room temperature for 1.5 h. Protein bands were visualized with ECL^TM^ Prime Western Blotting Detection Reagent (Amersham, GE Healthcare). Gel images were obtained employing the MiniChemi professional machine (SageCreation Science Co., Ltd., Beijing, China). The semi-quantification of protein bands was performed using ImageJ software.

### Chromatin Immunoprecipitation (ChIP)

A Magna CHIP^TM^ A chromatin immunoprecipitation kit (Millipore) was used according to the manufacturer’s protocol. Briefly, WiT49 cells were treated with 5 μM CHIR99021 for 48 h, and then the cells (5.4 × 10^6^/T75) were fixed with 1% formaldehyde for 20 min at room temperature. Sonication was performed for 5 min (with cycles of 30 s on/30 s off) in a Bioruptor^®^ Pico (Diagenode) on 300 μl cell lysates in 1.5-ml Bioruptor Pico microtubes with Caps (Diagenode, Cat. No. C30010016) at a concentration of 18 × 10^6^ cells/ml of nuclear lysis buffer. Acetyl-Histone 3 and Rabbit IgG in the Magna CHIP^TM^ A kit were used as positive and negative controls, respectively. Mouse Pol II antibody from the Magna CHIP^TM^ G kit (Millipore) was also used as a control. Rabbit polyclonal anti-β-catenin (Cat. 51067-2-AP, Proteintech) was used. The DNA yielded was then amplified by PCR with primers specific to different regions, using HotStarTaq Plus DNA polymerase (Qiagen) with the primers shown in Table 1. The PCR temperature program for these ChIP DNA samples is 95°C for 5 min, 40 cycles of 94°C for 30 s, 58°C for 30 s, and 72°C for 35 s, followed by an extension step of 72°C for 5 min. ChIP PCR products were loaded to the 1.5% agarose gel, and electrophoresis was performed.

### Luciferase Assay

FuGENE^®^ HD transfection reagent (Roche) and luciferase assay system with reporter lysis buffer (Promega) were used for the promoter transcriptional activity assay according to the manufacturer’s instructions. Briefly, 10^5^ cells were seeded to each well of a 24-well plate 18–20 h before transfection and maintained in 1 ml media. Serum-free DMEM media was mixed with FuGENE^®^ HD, followed by incubation at room temperature for 5 min. Plasmids were then added to this mixture and mixed, followed by incubation for 20 min. The ratio between the FuGENE^®^ HD volume and total plasmid quantity was 3 μl:1 μg. A volume of 40 μl of the serum-free DMEM/FuGENE^®^ HD/plasmids mixture was added to each well. Cells were harvested 48 h after transfection using 100 μl of 1 × reporter lysis buffer in each well of the 24-well plate on a Belly Dancer (Stovall Life Science, Inc) for 20 min at room temperature. Luciferase assay was performed by adding 10 μl of cell lysate to 50 μl of lyophilized luciferase assay reagent II (LAR II) (Promega) and tapping the reaction mixture for 10 s. The reaction was then measured by a Modulus^TM^ single tube multimode reader (Turner Biosystems). The renilla activity of the same tube was also measured. The value of luciferase activity relative to the renilla activity of the same sample was used for data analysis. Student’s *t*-tests (two tails, unpaired) were performed using Excel software (Microsoft), with *p* < 0.05 considered statistically significant.

### Immunofluorescence

Sterile coverslips were placed in 6-well plates before WiT49 cell seeding at a density of 100,000 cells/cm^2^. Cells were allowed to grow for 2 days before fixing with 8% paraformaldehyde in 1 × PBS (pH 7.0, 500 μl/well) for 15 min. The fixation was stopped with 100 mM glycine, followed by 0.1% Triton X-100 treatment. Cells were blocked with 1% BSA (Sigma, 2 ml/well) in PBS (pH 7.5) for 30 min at room temperature. Afterward, the coverslip was lifted, and the side with cells attached was placed face down directly in contact with 100 μl of primary antibody (1/200 diluted) on plastic at 4°C overnight. The coverslips were then incubated with 100 μl of secondary antibodies (1/200 diluted) in 1% BSA dissolved in PBS (pH 7.5) at room temperature for 60 min in the dark. Anti-rabbit secondary antibody AlexaFluor 546 (red) F(ab)_2_ GαR (Invitrogen) or anti-mouse secondary antibody AlexaFluor 488 (green) F(ab)_2_ GαM (Invitrogen) was used. Afterward, the cells on each coverslip were stained with ProLong Gold (Invitrogen), which contains 4′-6-diamidino-2-phenylindole (DAPI). The glass slides were examined under an Olympus BX51 fluorescent microscope and analyzed.

### Immunohistochemistry (IHC) and Hematoxylin and Eosin (H&E) Staining

Histology was performed to quantify *Aldh1a2* and *Ctnnb1* (β-catenin) expression in paraffin-embedded rat E18.5 embryonic kidney samples. IHC was performed on sections using rabbit polyclonal anti-β-catenin (Cat. 51067-2-AP, Proteintech) and rabbit polyclonal anti-ALDH1A2 (Cat. ab75674, Abcam). H&E staining was performed using Mayer’s hematoxylin solution.

### Software

Primers were designed using the PrimerQuest Design Tool^1^.

### Statistical Analysis

Student’s *t*-test was used to compare the significance of the difference between every two groups. Statistical analysis was performed using Excel software (Microsoft). Results were presented as mean ± standard error of the mean. All *P*-values were two-tailed, and *P* < 0.05 was considered to be a statistically significant difference.

## Results

### GSK3 Inhibitor CHIR99021 Inhibits *Aldh1a2* Expression in WiT49 Cells

**FIGURE 1:**
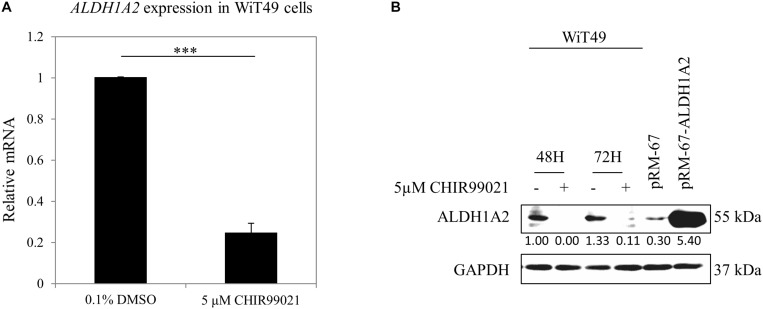
GSK3 inhibitor CHIR99021 inhibits *Aldh1a2* expression in WiT49 cells. **(A)** Effect of CHIR99021 on ALDH1A2 mRNA expression in WiT49 cells. WiT49 cells were treated with 0.1% DMSO (control vehicle) or 5 μM CHIR99021 for 48 h. *ALDH1A2* expression relative to *TBP* was measured by real-time RT-PCR. Values are the mean ± SD of three determinations; representative results from at least three separate experiments. ^∗∗∗^*P* < 0.001 compared with the DMSO control group (without CHIR99021 addition). **(B)** Effect of CHIR99021 on ALDH1A2 protein expression in WiT49 cells. WiT49 cells were treated with 0.1% DMSO or 5 μM CHIR99021 for 48 or 72 h. ALDH1A2 and GAPDH protein expression were measured by Western blot. pRM-67-ALDH1A2, positive control of ALDH1A2 protein made by transfection of mammalian expression vector pReceiver-M67-ALDH1A2 to WiT49 cells. pRM-67, negative control of ALDH1A2 protein generated by transfection of pReceiver-M67 empty vector. Values under protein bands were calculated by the density of bands of ALDH1A2 divided by the density of bands of GAPDH.

Previous studies have used CHIR99021 in a range of 1–10 μM in experiments with fetal kidney cells (Francipane and Lagasse, 2015) or kidney organoids generated from iPSC (Takasato et al., 2016; Taguchi and Nishinakamura, 2017; Przepiorski et al., 2018). We used a concentration of 5 μM, which is the median value of the concentration range that has been used in these experiments. WiT49 cells were treated with 0.1% DMSO or 5 μM CHIR99021 for 48 h. Real-time PCR results show that *ALDH1A2* mRNA levels were reduced to 25% of that measured in DMSO control cultures (Figure 1A). Western blot shows that ALDH1A2 protein levels were also significantly repressed in the CHIR99021 group compared to the DMSO control (Figure 1B).

### Inhibition of *ALDH1A2* Expression by GSK3 Inhibitor CHIR99021 Is via β-Catenin Signaling

**FIGURE 2:**
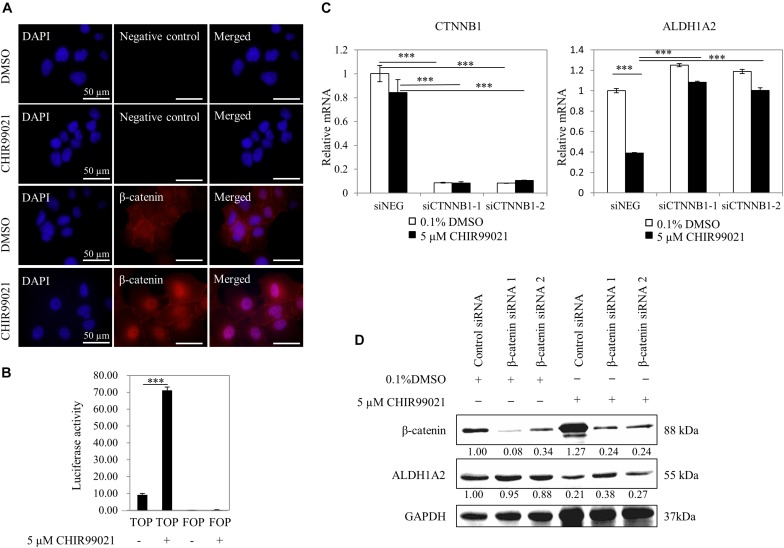
Inhibition of *ALDH1A2* expression by GSK3 inhibitor CHIR99021 is via β-catenin signaling. **(A)** Effect of CHIR99021 on β-catenin nuclear translocation in WiT49 cells. WiT49 cells were treated with 5 μM CHIR99021 or 0.1% DMSO for 48 h, and then immunofluorescence was performed with β-catenin antibody and DAPI staining. Negative control represents no primary antibody control. Bar length equals 50 μm. **(B)** Effect of CHIR99021 on the TCF reporter in WiT49 cells. A 500-ng sample of TOPFlash or FOPFlash constructs, together with 50 ng of the pRL-TK renilla control vector, was transiently transfected into WiT49 cells. Cells were treated with 0.1% DMSO or 5 μM CHIR99021 after 24 h of transfection. Luciferase reporter activities relative to renilla (pRL-TK) are shown in percentage terms. Values are the mean ± SD of three determinations from separate experiments. ^∗∗∗^*P* < 0.001 compared with the DMSO control group (without CHIR99021 addition). **(C,D)** Effect of *CTNNB1* knockdown on the downregulation of ALDH1A2 by CHIR99021 treatment. WiT49 was transfected with 50 nM CTNNB1 siRNA or negative control siRNA for 24 h, followed by treatment of 5 μM CHIR99021 for another 48 h. CTNNB1 and ALDH1A2 mRNA expression relative to TBP control in WiT49 cells were measured by real-time RT-PCR **(C)**. Values are the mean ± SD of three determinations from separate experiments. ^∗∗∗^*P* < 0.001 compared with the siNEG control group. β-catenin (CTNNB1), ALDH1A2 and GAPDH protein expression in WiT49 cells were measured by Western blot **(D)**. Values under bands were calculated by the density of bands of β-catenin or ALDH1A2 divided by the density of bands of GAPDH.

To confirm that the GSK3 inhibitor CHIR99021 affects Wnt/β-catenin signaling, we measured whether CHIR99021 affects the β-catenin nuclear translocation and the activity of TCF reporter. WiT49 cells were treated with 0.1% DMSO or 5 μM CHIR99021 for 48 h. Immunofluorescence detection of β-catenin shows that β-catenin protein mainly presented on the membrane/cytoplasm of WiT49 cells of the 0.1% DMSO control. With 5-μM CHIR99021 treatment, the majority of β-catenin translocated to the nucleus of WiT49 cells (Figure 2A). Luciferase assay results show that CHIR99021 activated TOPFlash TCF reporter (Figure 2B), suggesting that CHIR99021 treatment can activate Wnt/β-catenin signaling in WiT49 cells.

Next, we tested whether repression of *ALDH1A2* by CHIR99021 requires β-catenin. The expression of *CTNNB1* (β-catenin) in WiT49 cells was knocked down with siRNA for 24 h, followed by treatment of cells with 0.1% DMSO or 5 μM CHIR99021 for another 48 h. Real-time RT-PCR shows that when *CTNNB1* was knocked down, CHIR99021 failed to represses *ALDH1A2* (Figure 2C). Suppression of *CTNNB1* alone slightly upregulated *ALDH1A2* expression (Figure 2C), probably because β-catenin is mainly located on the membrane/cytoplasm in WiT49 cells. Western blot shows that in the 0.1% DMSO control group, *CTNNB1* knockdown had little effect on *ALDH1A2* expression. In the 5-μM CHIR99021 treatment group, *CTNNB1* knockdown led to *ALDH1A2* upregulation (Figure 2D). These data suggest that the repression of *ALDH1A2* expression by CHIR99021 requires CTNNB1. Taken together, these results suggest that canonical Wnt signaling represses *ALDH1A2* expression.

### Distinct Wnt Family Members Can Repress *ALDH1A2* Expression in WiT49 Cells

**FIGURE 3:**
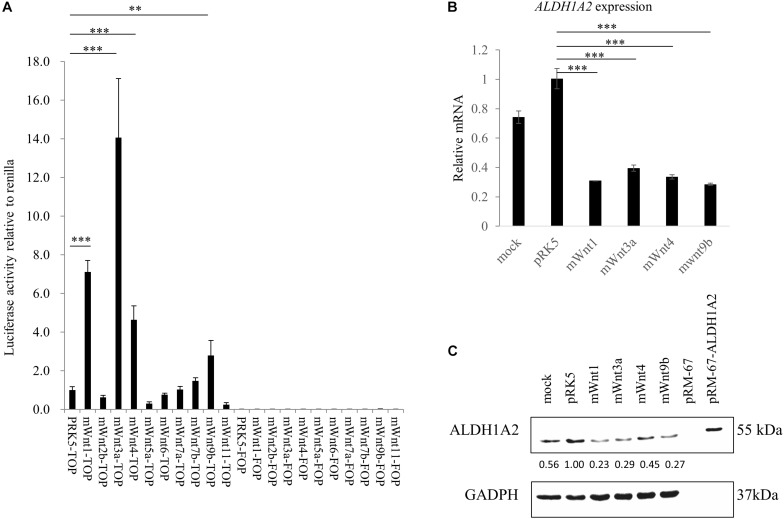
Wnts affect *ALDH1A2* expression in WiT49 cells. **(A)** Effects of Wnts on TCF reporter activity in WiT49 cells. 400 ng TOPFlash or FOPFlash constructs with 100 ng pRK5-mWnts expression vectors or pRK-5 empty vector, as well as 25 ng of the pRL-TK renilla control vector, was transiently transfected into WiT49 cells for 48 h. The pRK5-mWnts include pRK5-mWnt1, pRK5-mWnt2b, pRK5-mWnt3a, pRK5-mWnt4, pRK5-mWnt5a, pRK5-mWnt6, pRK5-mWnt7a, pRK5-mWnt7b, pRK5-mWnt9b, and pRK5-mWnt11. Luciferase reporter activities relative to renilla (pRL-TK) are shown. Values are the mean ± SD of four determinations; representative results from at least three separate experiments are shown. ^∗∗^*P* < 0.01 and ^∗∗∗^*P* < 0.001 compared with their respective controls, calculated using the Student’s *t*-test. **(B,C)** Effect of Wnts on *ALDH1A2* expression in WiT49 cells. WiT49 cells were transiently transfected with pRK5-mWnt1, pRK5-mWnt3a, pRK5-mWnt4, pRK5-mWnt9b, or the empty vector pRK5 for 48 h. The *ALDH1A2* expression relative to TBP was measured by real-time RT-PCR **(B)**. Values are the mean ± SD of three determinations; representative results from at least three separate experiments. ^∗∗^*P* < 0.01 and ^∗∗∗^*P* < 0.001 compared with the empty vector pRK5 control group. The ALDH1A2 protein expression was measured by Western blot **(C)**. pRM-67-ALDH1A2, positive control of ALDH1A2 protein made by transfection of mammalian expression vector pReceiver-M67-ALDH1A2 to WiT49 cells. pRM-67, negative control of ALDH1A2 protein generated by transfection of pReceiver-M67 empty vector. Per lane, 50 μg of the sample was loaded, and 2 μg ALDH1A2 protein-positive control was loaded. Mock represents samples of cells treated with transfection reagent and without plasmid DNA. Values under bands were calculated by the density of bands of ALDH1A2 divided by the density of bands of GAPDH.

Before measuring whether Wnt molecules may affect *ALDH1A2* expression, we tested whether Wnts that have been shown to express or have an effect in kidney development (Halt and Vainio, 2014) may activate the TCF reporter. Luciferase assay using TOPFlash/FOPFlash reporter shows that transient transfection of mouse *Wnt1*, *Wnt3a*, *Wnt4*, and *Wnt9b* expression vector to WiT49 cells for 48 h led to significant activation of the TOPFlash reporter, suggesting that these four Wnts can activate the canonical Wnt signaling in WiT49 cells. Six other mouse Wnts, namely Wnt2b, Wnt5a, Wnt6, Wnt7a, Wnt7b, and Wnt11, did not affect TOPFlash reporter activity (Figure 3A).

Next, we transiently transfected expression vectors of Wnts that have been shown to activate TCF reporter (Figure 3A) to WiT49 cells and measured whether *ALDH1A2* expression was altered. Mouse *Wnt1*, *Wnt3a*, *Wnt4*, and *Wnt9b* expression vectors were transiently transfected to WiT49 cells for 48 h. Samples were tested for *ALDH1A2* expression. Real-time RT-PCR and Western blot results both show that mouse *Wnt1*, *Wnt3a*, *Wnt4*, and *Wnt9b* transfection led to repression of *ALDH1A2* expression (Figures 3B,C).

Taken together, the above data suggest that Wnt/β-catenin signaling can repress *ALDH1A2* expression in the fetal kidney model WiT49 cells.

### β-Catenin and Aldh1a2 Proteins Are Localized in Distinct, Adjacent Domains *in vivo*

**FIGURE 4:**
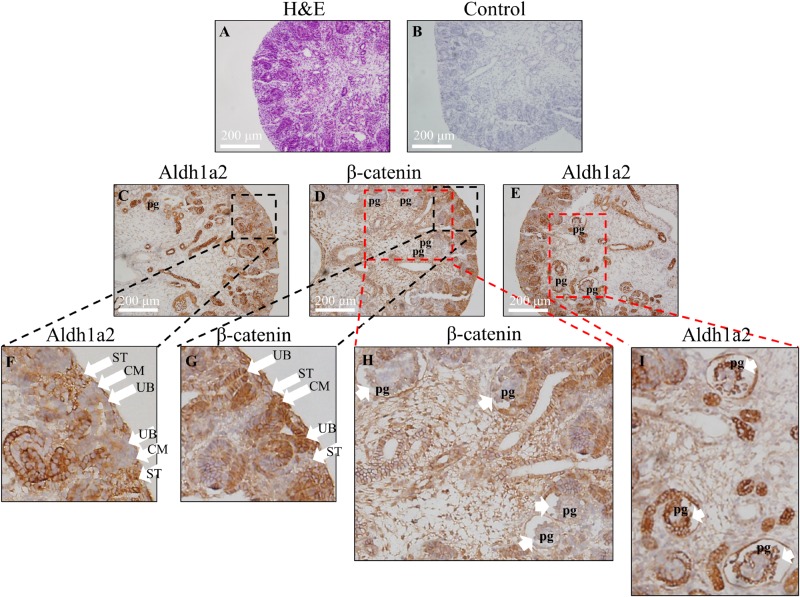
Inverse correlation of *Aldh1a2* localization with β-catenin in E18.5 embryonic rat kidney. **(A)** Hematoxylin and eosin (H&E) staining of rat embryonic kidney (E18.5). Rat embryonic kidney (E18.5) were immunohistochemically stained with no antibody control **(B)**, ALDH1A2 antibody **(C,E,F,I),** and β-catenin antibody **(D,G,H)**. CM, cap mesenchyme; UB, ureteric bud; ST, stroma; pg, the primitive podocytes of the glomerulus. Length of the bar equals 200 μm.

Having shown negative regulation of *ALDH1A2* expression by β-catenin in WiT49 cell line *in vitro*, we next address whether the localization pattern of *Aldh1a2* and β-catenin in the fetal kidney is consistent with this repressive effect. We determined the Aldh1a2 and β-catenin protein localization in the rat E18.5 fetal kidney by immunohistochemistry. The results show that *Aldh1a2* primarily localized in the stroma of the developing renal cortex but did not localize in the ureteric bud and cap mesenchyme (Figures 4C,F). β-catenin strongly localized in the ureteric bud and cap mesenchyme, but little was detectable in the developing stroma (Figures 4D,G). In the developing podocytes, *Aldh1a2* was strongly localized (Figures 4E,I), while β-catenin was not detectable (Figures 4D,H). These data suggest an inverse relationship between β-catenin and *Aldh1a2* protein localization in the fetal kidney.

### β-Catenin Is Associated With *ALDH1A2* Promoter and Intron1G

**FIGURE 5:**
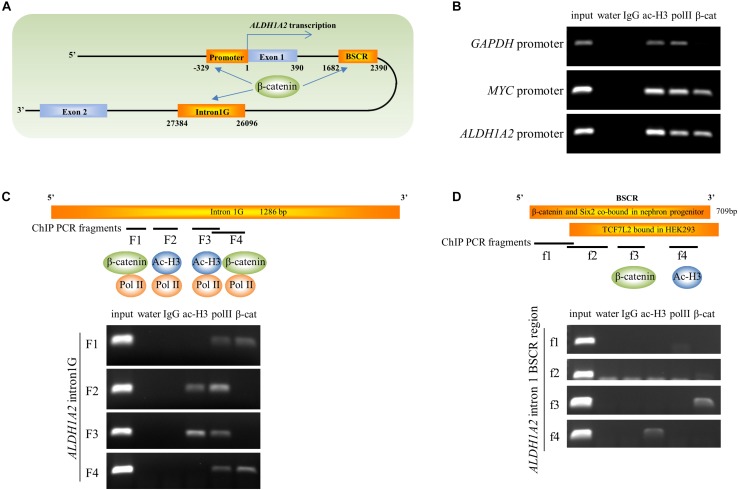
β-catenin is associated with the *ALDH1A2* gene promoter and intron1G enhancer. **(A)** Diagram of 5′ terminal of the *ALDH1A2* gene. DNA elements include *ALDH1A2* promoter, intron1G, β-catenin, and the Six2 co-bound region (BSCR). **(B)** Chromatin immunoprecipitation demonstrating the binding of β-catenin to the *ALDH1A2* promoter in WiT49 cells. β-catenin is associated with the promoter of the known β-catenin target *C-MYC* but not with the promoter of the housekeeping gene *GAPDH*. Acetyl histone 3 (ac-H3) and RNA polymerase II (pol II) were associated with all three gene promoters. **(C)** Chromatin immunoprecipitation demonstrating binding of β-catenin to the evolutionarily conserved element intron1G within intron 1 of the *ALDH1A2* gene (Castillo et al., 2010) at two loci (F1 and F4) in WiT49 cells. **(D)** Chromatin immunoprecipitation showing binding of β-catenin to the f3 fragment of the “β-catenin and Six2 co-bound region (BSCR)” within intron 1 of the *ALDH1A2* gene in WiT49 cells. BSCR was previously shown to co-bound by β-catenin and Six2 in nephron progenitor (chr9:71065207–71065805) (Park et al., 2012). This region partially overlapped with a TCF7L2 (TCF4) bound region in HEK293 previously suggested by a ChIP-seq result (Frietze et al., 2012).

Given the ability of β-catenin to repress *ALDH1A2* expression, we next determined whether β-catenin is associated with the *ALDH1A2* promoter and two DNA elements within *ALDH1A2* intron 1 using ChIP analysis. One DNA element, named “intron1G,” was previously shown to activate *Aldh1a2* expression in the mouse spine (Castillo et al., 2010). Another DNA element, which we named the “β-catenin and Six2 co-bound region (BSCR),” was previously shown to recruit both β-catenin and Six2 in fetal kidney pretubular aggregate (Park et al., 2012) (Figure 5A). In our ChIP assay, control acetyl-histone 3 and pol II immunoprecipitations confirmed the presence of acetyl-histone 3 and RNA polymerase II at the promoter of *GAPDH* (Figure 5B) and also showed their recruitment to the *MYC* and *ALDH1A2* promoters (Figure 5B). β-catenin is known to regulate the *MYC* promoter, and *MYC* promoter DNA was precipitated by the β-catenin antibody (Figure 5B), providing a positive control. Significantly, the β-catenin antibody also precipitated the *ALDH1A2* promoter DNA (Figure 5B).

In the ChIP assay of the *ALDH1A2* intron1G DNA element, the presence of RNA polymerase II was confirmed by pol II immunoprecipitations at the F1, F2, F3, and F4 fragment of intron1G (Figure 5C). The intense presence of Pol II on intron1G suggests that this conserved DNA element is very likely to be involved in *ALDH1A2* transcription. Acetyl-histone 3 only binds to F2 and F3 sites of intron1G (Figure 5C). β-catenin antibody precipitated the F1 and F4 fragments of intron1G (Figure 5C).

In the ChIP assay of the *ALDH1A2* BSCR element, the Pol II antibody does not precipitate any fragment of this BSCR element (Figure 5D). Acetyl-histone 3 only binds to f4 pieces of the BSCR element (Figure 5D). β-catenin antibody precipitated the f3 fragment of the BSCR element (Figure 5D).

Taken together, ChIP analysis of *ALDH1A2* promoter, intron1G, and BSCR elements suggests that β-catenin represses *ALDH1A2* expression through direct recruitment of β-catenin to these DNA elements.

### Canonical Wnt Signaling Activated by CHIR99021 Inhibits the Transcriptional Activity of *ALDH1A2* Promoter and Intron1G Enhancer

**FIGURE 6:**
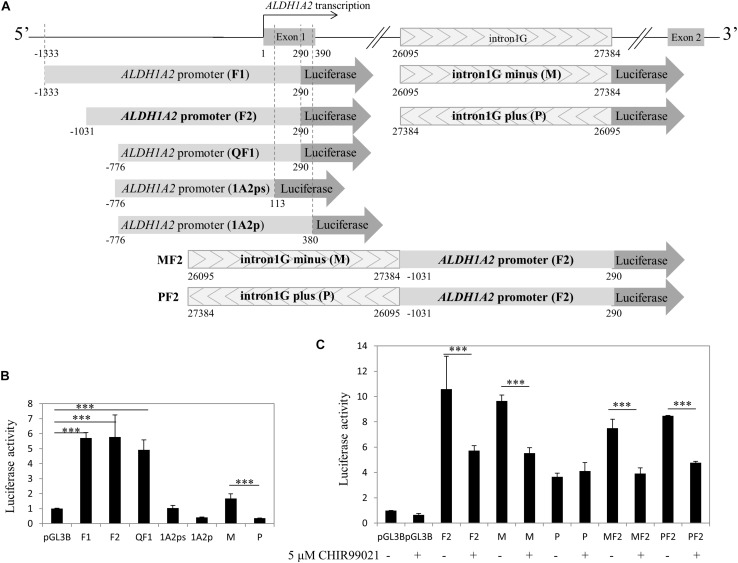
CHIR99021 represses *ALDH1A2* promoter and the intron1G element. **(A)** Maps of *ALDH1A2* promoter and intron1G DNA element, numbered relative to the *ALDH1A2* transcriptional start. pGL3B luciferase assay reporter driven by *ALDH1A2* promoter with different lengths (F1, F2, QF1, 1A2ps, 1A2p), intron1G with opposite orientations (M, P), and the combination of intron1G and *ALDH1A2* promoter (MF2, PF2). **(B)** Identification of the essential promoter region of the *ALDH1A2* gene. A 500-ng sample of pGL3-basic (pGL3B) or of pGL3B inserted with *ALDH1A2* promoter of different lengths, together with 25 ng of the pRL-TK renilla control vector, was transiently transfected to HEK293 cells for 48 h. Luciferase reporter activities relative to renilla control are shown. Values are the mean ± SD of three determinations; representative results from at least three separate experiments. ^∗∗∗^*P* < 0.001 compared with the empty vector pGL3B control group. **(C)** The repressive effect of CHIR99021 to *ALDH1A2* promoter and intron1G. A 500-ng sample of pGL3B, pGL3B construct driven by *ALDH1A2* promoter, intron1G, or the combination of intron1G and *ALDH1A2* promoter was transiently transfected to WiT49 cells. At 24 h of transfection, cells were treated with 0.1% DMSO or 5 μM CHIR99021 for another 48 h. Luciferase reporter activities relative to renilla (pRL-TK) are shown. Values are the mean ± SD of four determinations; representative results from at least three separate experiments. ^∗∗∗^*P* < 0.001 compared with their respective controls, calculated using the Student’s *t*-test.

Given that both the control acetyl-histone 3 and pol II antibodies immunoprecipitated *ALDH1A2* promoter and multiple sites of intron1G, chromatin within these two sites is more likely to be in an open state and relevant to the *ALDH1A2* transcription. To examine whether canonical Wnt signaling activated by CHIR99021 may affect the transcriptional activity of the *ALDH1A2* promoter and intron1G element, we conducted luciferase assay. First, we cloned *ALDH1A2* promoter of different lengths, intron1G with opposite orientations, and the combination of intron1G and *ALDH1A2* promoter (Figure 6A). The luciferase assay results show that the *ALDH1A2* promoter F2 (−1031 bp to 290 bp relative to the transcription start site of NM_003888) is the most active and essential region for *ALDH1A2* promoter activation (Figure 6B). Comparison between *ALDH1A2* promoters QF1 and 1A2ps suggests that a region from 113 to 290 bp (relative to the transcription start site) is the basal and most essential element for *ALDH1A2* promoter activity (Figure 6B). Comparison between *ALDH1A2* promoter QF1 and 1A2p suggests that a region between 290 and 380 bp has a strong inhibitory effect on the *ALDH1A2* promoter activity. The luciferase activity of intron1G at minus strand (M) is higher than that of intron1G at the plus strand (P) (Figure 6B).

Since the above results show that β-catenin binds to the *ALDH1A2* promoter and enhancers within intron 1, we next used data mining on *in situ* Hi-C data (Rao et al., 2014) to see if these elements are within a chromatin loop, which is a chromatin structure enabling interaction of enhancer and promoter. We found that the *ALDH1A2* promoter, intron1G, and BSCR are within a chromatin loop defined by CTCF binding sites (Supplementary Figure S1). To explore whether *ALDH1A2* promoter, Intron1G, and BSCR sequences are in an open chromatin state, we aligned sequences of these three elements to the UCSC genome browser on human Feb. 2009 (GRCh37/hg19) assembly. The alignment of *ALDH1A2* promoter, Intron1G, and BSCR sequences to the UCSC genome browser shows that the *ALDH1A2* promoter and intron1G are relatively in an open chromatin state in multiple cell lines (Supplementary Figure S2) (Ernst et al., 2011), suggesting that they may potentially interact with each other. Although these results from data mining are not obtained from fetal kidney cells, they still provide the useful information that *ALDH1A2* promoter, intron1G, and BSCR may be within the same chromatin loop and in an open chromatin state, considering that chromatin 3D structure is usually conserved (Dixon et al., 2012; Rao et al., 2014).

Therefore, we combined the *ALDH1A2* promoter and intron1G to see their transcriptional activity and whether canonical Wnt signaling activated by CHIR99021 affects their transcriptional activity. Luciferase assay was performed to measure the effect of canonical Wnt signaling activated by CHIR99021 on the *ALDH1A2* promoter F2, intron1G with opposite orientations, or the combination of intron1G with F2. The luciferase assay results show that the canonical Wnt signaling activated by CHIR99021 represses the transcriptional activity of both the *ALDH1A2* promoter F2 and the intron1G in WiT49 cells (Figure 6C). The canonical Wnt signaling activated by CHIR99021 also suppresses the combination of intron1G and *ALDH1A2* promoter F2 (Figure 6C).

Taken together, the canonical Wnt signaling activated by CHIR99021 can repress the *ALDH1A2* promoter and intron1G DNA element.

## Discussion

The Wnt/β-catenin signaling and retinoic acid pathways are critical factors regulating fetal kidney development (Mendelsohn et al., 1999; Halt and Vainio, 2014). The retinoic acid synthetic enzyme ALDH1A2, which is essential for renal development (Rosselot et al., 2010), strongly expresses in the stroma of the renal outer cortex (Batourina et al., 2001; Niederreither et al., 2002; Marlier and Gilbert, 2004; Rosselot et al., 2010), while β-catenin predominantly expresses in the ureteric bud (Bridgewater et al., 2008; Marose et al., 2008; Wang et al., 2018), suggesting an inverse relationship between their expression in the fetal kidney. Recent data from several lines of evidence indicate that *ALDH1A2* could be a novel target of β-catenin signaling (Castillo et al., 2010; Frietze et al., 2012; Park et al., 2012; Hong et al., 2015). Indeed, we show here for the first time that activation of the canonical Wnt signaling pathway using CHIR99021 can negatively regulate *ALDH1A2* expression in WiT49 cells. We demonstrate that knockdown of *CTNNB1* abrogates the down-regulation of *ALDH1A2* by CHIR99021. Transient transfection of Wnt1, Wnt3a, Wnt4, and Wnt9b to WiT49 cells leads to *ALDH1A2* downregulation. We confirm that *Aldh1a2* and β-catenin expression in the fetal kidney are inversely correlated. Importantly, the *ALDH1A2* promoter and the intron1G element recruit β-catenin and are transcriptionally repressed by canonical Wnt signaling activated by CHIR99021, suggesting that β-catenin directly represses *ALDH1A2* expression via its promoter and intron1G. Taken together, we provide *in vitro* evidence showing that the Wnt/β-catenin pathway directly represses *ALDH1A2* expression in a fetal kidney cell model.

Although Wnt/β-catenin signaling is known to activate gene expression, the expression of several target genes can also be inhibited by the Wnt/β-catenin signaling (including *E-cadherin*, *Hath1*, *Sox9*, *15-PGDH, RANKL* and *Osteocalcin*) (Jamora et al., 2003; Kahler and Westendorf, 2003; Leow et al., 2004; Hill et al., 2005; Spencer et al., 2006; Smartt et al., 2012). It has also been shown that the GSK3 inhibitors (canonical Wnt signaling activator) can repress *PEPCK* and *G6Pase* gene expression (Lochhead et al., 2001). The exact mechanisms that are responsible for transcriptional repression are less well known than for transcriptional activation by Wnt/β-catenin signaling. As our understanding of the mechanisms of gene repression by β-catenin gets better, it will be of tremendous interest to elucidate the mechanism that was responsible for *ALDH1A2* suppression.

Our work shows that β-catenin is strongly localized in the ureteric bud of the E18.5 rat kidney and does not present in the developing stroma, consistent with previous reports showing that β-catenin and its signaling primarily present in the ureteric bud (Bridgewater et al., 2008; Marose et al., 2008; Wang et al., 2018). Other literature also shows that β-catenin intensely presents in the branching ureteric bud (Iglesias et al., 2007; Billfeldt et al., 2016; Kovacs et al., 2017; Terada et al., 2017). Although our immunohistochemistry result shows that β-catenin is mainly localized to the membrane/cytosol, another study has documented the nuclear staining of β-catenin in ureteric bud cells using immunofluorescence method (Wang et al., 2018). Active canonical WNT signaling (TCF reporter activity) was obvious in the branching ureteric bud throughout its transition into renal tubules (Iglesias et al., 2007). The presence of Wnt/β-catenin signaling in the ureteric bud is also supported by the fact that *Axin2*, the Wnt/β-catenin signaling typical target, expresses in the ureteric bud of the fetal kidney (Mohri et al., 2011). Taken together, it is clear that Wnt/β-catenin signaling presents in the ureteric bud, supported by all three types of evidence from the literature and our result (Figure 4), including expression of β-catenin/activated β-catenin, TCF reporter activity, and conditional knockout of β-catenin driven by the *Hoxb7* promoter (Iglesias et al., 2007; Bridgewater et al., 2008; Marose et al., 2008; Billfeldt et al., 2016; Kovacs et al., 2017; Terada et al., 2017; Wang et al., 2018).

Our result showing that β-catenin presents in the cap mesenchyme is consistent with previous reports showing that β-catenin expresses in Six2 positive cap mesenchyme cells (Park et al., 2007; Karner et al., 2011; Park et al., 2012), in Myc positive nephron progenitor cells (Pan et al., 2017), and in the nephrogenic mesenchyme (Iglesias et al., 2007; Billfeldt et al., 2016; Kovacs et al., 2017; Terada et al., 2017). Furthermore, active canonical WNT signaling (TCF reporter activity) was obvious in the epithelia of the nephrogenic mesenchyme (Iglesias et al., 2007). However, other works show an absence of β-catenin activity in nephron progenitor (Burn et al., 2011; Tanigawa et al., 2011). These works may lack evidence from the higher resolution pictures of β-catenin staining and TCF reporter activity on the cap mesenchyme that would be necessary to show a lack of β-catenin activity in cap mesenchyme. Taken together, it is very likely that cap mesenchyme also has β-catenin activity, considering that all three types of evidence present, namely the intense β-catenin staining, TCF reporter activity, and conditional knockout of β-catenin within *Six2* expression positive cells, result in a phenotype (Iglesias et al., 2007; Park et al., 2007; Karner et al., 2011; Park et al., 2012; Billfeldt et al., 2016; Kovacs et al., 2017; Pan et al., 2017; Terada et al., 2017).

Our result showing that β-catenin is not present in the developing podocyte is consistent with a previous report showing that TCF reporter activity is quickly downregulated in maturing nephrons and becomes undetectable in the postnatal kidney (Iglesias et al., 2007).

Our work showing that β-catenin does not present in the developing stroma is also consistent with previous reports. However, it has been shown in other studies that β-catenin presents in the developing stroma (Yu et al., 2008; Boivin et al., 2015) and the medullar stroma of the developing human kidney (Billfeldt et al., 2016). This discrepancy may arise from differences in the specificity and sensitivity of different β-catenin antibodies and detection methods (IHC vs. immunofluorescence). Although conditional deletion of β-catenin in stroma driven by the *Foxd1* promoter shows a phenotype of kidney development (Yu et al., 2008; Boivin et al., 2015), most of the literature supports β-catenin not being expressed in the developing stroma (Iglesias et al., 2007; Park et al., 2007; Bridgewater et al., 2008; Marose et al., 2008; Karner et al., 2011; Park et al., 2012; Kovacs et al., 2017; Pan et al., 2017; Terada et al., 2017; Wang et al., 2018). The presence of Wnt/β-catenin signaling in stroma also needs support from result of TCF luciferase reporter activity (Yu et al., 2008; Boivin et al., 2015). At least we can be sure that Wnt/β-catenin expression/signaling in the ureteric bud and cap mesenchyme is stronger than that in the developing stroma.

We show that activation of Wnt/β-catenin signaling by CHIR99021 represses *ALDH1A2* expression. This result reveals for the first time that β-catenin can downregulate, rather than upregulate, *Aldh1a2* expression, indicating that the canonical Wnt signaling is upstream of retinoic acid synthesis. This finding is different from reports suggesting that Wnt/β-catenin signaling may upregulate *Aldh1a2* expression in Zebrafish development (Wehner et al., 2014) and dendritic cells (Suryawanshi and Manicassamy, 2015). This difference suggests that the regulation of *Aldh1a2* expression by β-catenin may be tissue and developmental context-dependent, which is a common phenomenon for transcriptional regulation (Ma, 2005; Essafi et al., 2011). It would be of interest in the future to study what may contribute to this opposite regulation of *Aldh1a2* expression by Wnt/β-catenin signaling in different contexts.

The result that Wnt1, Wnt3a, Wnt4, and Wnt9b activate TOPFlash activity in WiT49 cells (Figure 3A) is consistent with reports showing that Wnt3a and Wnt9b activate Wnt/β-catenin signaling (Dallosso et al., 2009; Karner et al., 2011; Pan et al., 2017). The mild activation of TCF reporter by Wnt4 shown by us (Figure 3A) is different from reports showing that Wnt4 does not activate TCF reporter in fetal kidney cells (Tanigawa et al., 2011; Billfeldt et al., 2016). This discrepancy may arise from the difference in the expression of Wnt receptors. Nonetheless, our results demonstrate that Wnt molecules, at least Wnt3a, Wnt9b, and Wnt1, can activate TCF reporter activity and repress *ALDH1A2* expression in WiT49 cells. As *Wnt9b* is expressed in ureteric buds, we speculate that Wnt9b might directly repress *Aldh1a2* expression via canonical Wnt signaling in the ureteric branching by autocrine, leading to a proper local concentration of retinoic acid.

We have defined the F2 region (−1031 to 290 bp) as the most active promoter region of the *ALDH1A2* gene (Figure 6B). We found a region (290–380 bp) that has an inhibitory effect on the *ALDH1A2* promoter activity, and another region (96–290 bp) has an essential effect on *ALDH1A2* promoter activity (Figure 6B). This study also shows that *Aldh1a2* intron1G, which was shown to have an enhancing effect on the expression of *Aldh1a2* in the developing neuron cells (Castillo et al., 2010), can be repressed by activation of the canonical Wnt signaling pathway using CHIR99021 in WiT49 cells.

ChIP-PCR data show that pol II was recruited to all four regions within intron1G (Figure 5C), suggesting that this conserved element is very likely to be involved in pol II-dependent *ALDH1A2* transcription, although the intron1G is 26095 bp away from the *ALDH1A2* transcription start site. The basal luciferase activity of intron1G on the minus strand (M) is higher than that of intron1G on the plus strand (P) (Figure 6B). The luciferase assay result shows that the repressive effect of intron1G by CHIR99021 works on the minus strand (M) but not on the plus strand (P) (Figure 6C), suggesting that intron1G on the same stand of the *ALDH1A2* gene is more likely to play a role in the repressive regulation by β-catenin. The BSCR element is a DNA region previously suggested to be co-bound by β-catenin and Six2 in nephron progenitor (Park et al., 2012) and bound by TCF7L2 in HEK293 (Frietze et al., 2012) based on ChIP-seq data. We show by ChIP-PCR that β-catenin is recruited to the BSCR element at the f3 region, where acetyl-histone 3 and pol II do not bind (Figure 5D), suggesting that the chromatin state of this region may not be as open as that of intron1G.

Taken together, our data suggest a novel role for Wnt/β-catenin signaling in direct repression of *Aldh1a2* expression via the *Aldh1a2* promoter and the intron1G conserved element, which may lead to alteration of retinoic acid synthesis in ureteric bud branching and cap mesenchyme. Because *Aldh1a2*^–/–^ mutant mouse kidney exhibits a significant reduction of ureteric buds and nephrons (Rosselot et al., 2010) and excessive retinoic acid is teratogenic (Fantel et al., 1977; Lee et al., 2012; Piersma et al., 2017), our findings suggest that the regulation of *Aldh1a2* by Wnt/β-catenin signaling may play a significant role in the limitation of retinoic acid production in ureteric bud and cap mesenchyme, where a proper control of retinoic acid concentration is critical for renal development.

## Conclusion

Our findings of the direct repression of *Aldh1a2* by Wnt/β-catenin signaling have a significant consequence for the regulation of retinoic acid synthesis, which may play roles in fetal kidney development. Future study is necessary to address how the *Aldh1a2* expression and control of retinoic acid concentration are altered in the ureteric bud when *Ctnnb1* is conditionally knocked out in ureteric bud of fetal kidney in animal models.

## Data Availability Statement

The raw data supporting the conclusions of this article will be made available by the authors, without undue reservation, to any qualified researcher.

## Ethics Statement

The animal study was reviewed and approved by the Ethics Committee of Huazhong University of Science and Technology Union Shenzhen Hospital.

## Author Contributions

YL, JfD, and JhD designed and co-directed the research and wrote the manuscript. YL, HG, FZ, JZ, SL, and JfD performed the experiments, with the help of LW, JhD, and BZ in all assays involving cell cultures, and contributed to the interpretation of data. JW provided the pregnant rat kidney. HG, YL, FZ, JZ, and SL contributed to the immunohistochemistry experiment. JL provided reagents and knowledge of vitamin metabolisms. YL, JfD, HG, FZ, JhD, JW, JZ, SL, JL, LW, and BZ reviewed and contributed to manuscript editing. All authors read and approved the manuscript.

## Conflict of Interest

The authors declare that the research was conducted in the absence of any commercial or financial relationships that could be construed as a potential conflict of interest.

## Footnotes

1 http://www.idtdna.com/primerquest/Home/Index

## Supplementary Material

FIGURE S1 CCCTC-binding factor (CTCF) binding to *ALDH1A2* gene locus and formation of DNA loops in GM12878 cells. The arrows show the two distal DNA sites where the chromatin loops bind to each other (the straight line extending from the point directs to two DNA sites; in most cases, the CTCF binding site). The red (a, b, c) and blue (d, e) arcs indicate the extent of each chromatin loop, and the red chromatin loop (a, b, c) totally or partially covers the *ALDH1A2* gene. This figure was based on a previous report using the Hi-C method (Rao et al., 2014). The report detailed a Hi-C experiment conducted to examine the genome-wide chromatin loop in the GM12878 cell line. The data were integrated with the ChIP-seq data of CTCF, H3K36me3 (active mark), H3K27ac (active mark), H3K27me3 (repressive mark), H3K4me3 (active mark), H3K9me3 (repressive mark), H3K9ac (active mark), and Dnase Seq data. Since the chromatin loop is relatively stable among species and different tissues, the data of chromatin loop at *ALDH1A2* locus obtained from the lymphoblastoid cell line GM12878 give us clues on how the chromatin at *ALDH1A2* gene locus is organized. The data show that *ALDH1A2* promoter, intron1G, and the BSCR element are likely to be within the same chromatin loop. Thus, they may have close spatial proximity and less physical segmentation, suggesting that they have the potential to interact with each other and may be relevant to β-catenin recruitment and regulation of *ALDH1A2* expression.

FIGURE S2 The chromatin state of the *ALDH1A2* gene. Intron1G, BSCR, and *ALDH1A2* promoter sequences were searched on genome on the UCSC browser and highlighted in a light blue color. The lane “100 vertebrates Basewise Conservation by PhyloP” shows that the *ALDH1A2* promoter, intron1G, and BSCR element are all evolutionally conserved. The lane “Chromatin State Segmentation by HMM from ENCODE/Broad” shows that *ALDH1A2* promoter and intron1G are in an open chromatin state in nine cell lines, namely GM12878, H1-hESC, K562, HepG2, HUVEC, HMEC, HSMM, NHEK, and NHLF. The lane “DNaseI Hypersensitivity Clusters in 125 cell types from ENCODE (V3)” also shows that the *ALDH1A2* promoter and intron1G are in an open chromatin state. The lane “Transcription Factor ChIP-seq Clusters (161 factors) from ENCODE with Factorbook Motifs” shows moderate TCF7L2 binding on the intron1G and BSCR site.

## References

[B1] Alami J. Williams B. R. Yeger H. (2003). Derivation and characterization of a Wilms’ tumour cell line, WiT 49. *Int. J. Cancer* 107 365–374. 10.1002/ijc.11429 14506735

[B2] Batourina E. Gim S. Bello N. Shy M. Clagett-Dame M. Srinivas S. (2001). Vitamin A controls epithelial/mesenchymal interactions through Ret expression. *Nat. Genet.* 27 74–78. 10.1038/83792 11138002

[B3] Billfeldt N. K. Banyai D. Kovacs G. (2016). Absence of canonical WNT signaling in adult renal cell tumors of embryonal origin. *Anticancer Res.* 36 2169–2173. 27127119

[B4] Boivin F. J. Sarin S. Lim J. Javidan A. Svajger B. Khalili H. (2015). Stromally expressed beta-catenin modulates Wnt9b signaling in the ureteric epithelium. *PLoS One* 10:e0120347. 10.1371/journal.pone.0120347 25803581 PMC4372213

[B5] Bridgewater D. Cox B. Cain J. Lau A. Athaide V. Gill P. S. (2008). Canonical WNT/β-catenin signaling is required for ureteric branching. *Dev. Biol.* 317 83–94. 10.1016/j.ydbio.2008.02.010 18358465

[B6] Burn S. F. Webb A. Berry R. L. Davies J. A. Ferrer-Vaquer A. Hadjantonakis A. K. (2011). Calcium/NFAT signalling promotes early nephrogenesis. *Dev. Biol.* 352 288–298. 10.1016/j.ydbio.2011.01.033 21295565 PMC3070816

[B7] Carroll T. J. Park J. S. Hayashi S. Majumdar A. Mcmahon A. P. (2005). Wnt9b plays a central role in the regulation of mesenchymal to epithelial transitions underlying organogenesis of the mammalian urogenital system. *Dev. Cell* 9 283–292. 10.1016/j.devcel.2005.05.016 16054034

[B8] Castillo H. A. Cravo R. M. Azambuja A. P. Simões-Costa M. S. Sura-Trueba S. Gonzalez J. (2010). Insights into the organization of dorsal spinal cord pathways from an evolutionarily conserved raldh2 intronic enhancer. *Development* 137 507–518. 10.1242/dev.043257 20081195 PMC4074295

[B9] Cullen-Mcewen L. Sutherland M. R. Black M. J. (2016). “Chapter 3 – The human kidney: parallels in structure, spatial development, and timing of nephrogenesis,” in *Kidney Development, Disease, Repair and Regeneration*, ed. Little M. H. (San Diego: Academic Press), 27–40.

[B10] Dallosso A. R. Hancock A. L. Szemes M. Moorwood K. Chilukamarri L. Tsai H. H. (2009). Frequent long-range epigenetic silencing of protocadherin gene clusters on chromosome 5q31 in Wilms’ tumor. *PLoS Genet.* 5:e1000745. 10.1371/journal.pgen.1000745 19956686 PMC2776977

[B11] Davidson A. (2008). *Mouse Kidney Development.* Available at: www.stembook.org [accessed April 7, 2015].

[B12] Dixon J. R. Selvaraj S. Yue F. Kim A. Li Y. Shen Y. (2012). Topological domains in mammalian genomes identified by analysis of chromatin interactions. *Nature* 485 376–380. 10.1038/nature11082 22495300 PMC3356448

[B13] Dolle P. (2009). Developmental expression of retinoic acid receptors (RARs). *Nucl. Recept. Signal.* 7:e006. 10.1621/nrs.07006 19471585 PMC2686085

[B14] Dressler G. R. (2009). Advances in early kidney specification, development and patterning. *Development* 136 3863–3874. 10.1242/dev.034876 19906853 PMC2778737

[B15] Duester G. (2008). Retinoic acid synthesis and signaling during early organogenesis. *Cell* 134 921–931. 10.1016/j.cell.2008.09.002 18805086 PMC2632951

[B16] Ernst J. Kheradpour P. Mikkelsen T. S. Shoresh N. Ward L. D. Epstein C. B. (2011). Mapping and analysis of chromatin state dynamics in nine human cell types. *Nature* 473 43–49. 10.1038/nature09906 21441907 PMC3088773

[B17] Essafi A. Webb A. Berry R. L. Slight J. Burn S. F. Spraggon L. (2011). A Wt1-controlled chromatin switching mechanism underpins tissue-specific Wnt4 activation and repression. *Dev. Cell* 21 559–574. 10.1016/j.devcel.2011.07.014 21871842 PMC3604688

[B18] Fan X. Molotkov A. Manabe S.-I. Donmoyer C. M. Deltour L. Foglio M. H. (2003). Targeted disruption of Aldh1a1 (Raldh1) provides evidence for a complex mechanism of retinoic acid synthesis in the developing retina. *Mol. Cell. Biol.* 23 4637–4648. 10.1128/mcb.23.13.4637-4648.2003 12808103 PMC164835

[B19] Fantel A. G. Shepard T. H. Newell-Morris L. L. Moffett B. C. (1977). Teratogenic effects of retinoic acid in pigtail monkeys (*Macaca nemestrina*) I, General features. *Teratology* 15 65–71. 10.1002/tera.1420150109 402705

[B20] Fetting J. L. Guay J. A. Karolak M. J. Iozzo R. V. Adams D. C. Maridas D. E. (2013). FOXD1 promotes nephron progenitor differentiation by repressing decorin in the embryonic kidney. *Development* 141 17–27. 10.1242/dev.089078 24284212 PMC3865747

[B21] Francipane M. G. Lagasse E. (2015). The lymph node as a new site for kidney organogenesis. *Stem Cells Transl. Med.* 4 295–307. 10.5966/sctm.2014-0208 25646529 PMC4339853

[B22] Frietze S. Wang R. Yao L. Tak Y. G. Ye Z. Gaddis M. (2012). Cell type-specific binding patterns reveal that TCF7L2 can be tethered to the genome by association with GATA3. *Genome Biol.* 13:R52. 10.1186/gb-2012-13-9-r52 22951069 PMC3491396

[B23] Grun F. Hirose Y. Kawauchi S. Ogura T. Umesono K. (2000). Aldehyde dehydrogenase 6, a cytosolic retinaldehyde dehydrogenase prominently expressed in sensory neuroepithelia during development. *J. Biol. Chem.* 275 41210–41218. 10.1074/jbc.M007376200 11013254

[B24] Halt K. Vainio S. (2014). Coordination of kidney organogenesis by Wnt signaling. *Pediatr. Nephrol.* 29 737–744. 10.1007/s00467-013-2733-z 24445433 PMC3928513

[B25] Hill T. P. Später D. Taketo M. M. Birchmeier W. Hartmann C. (2005). Canonical Wnt/β-catenin signaling prevents osteoblasts from differentiating into chondrocytes. *Dev. Cell* 8 727–738. 10.1016/j.devcel.2005.02.013 15866163

[B26] Hong Y. Manoharan I. Suryawanshi A. Majumdar T. Angus-Hill M. L. Koni P. A. (2015). Beta-catenin promotes regulatory T-cell responses in tumors by inducing vitamin A metabolism in dendritic cells. *Cancer Res.* 75 656–665. 10.1158/0008-5472.can-14-2377 25568183 PMC4333068

[B27] Iglesias D. M. Hueber P. A. Chu L. Campbell R. Patenaude A. M. Dziarmaga A. J. (2007). Canonical WNT signaling during kidney development. *Am. J. Physiol. Renal Physiol.* 293 F494–F500. 10.1152/ajprenal.00416.2006 17494089

[B28] Itaranta P. Lin Y. Perasaari J. Roel G. Destree O. Vainio S. (2002). Wnt-6 is expressed in the ureter bud and induces kidney tubule development in vitro. *Genesis* 32 259–268. 10.1002/gene.10079 11948913

[B29] Jamora C. Dasgupta R. Kocieniewski P. Fuchs E. (2003). Links between signal transduction, transcription and adhesion in epithelial bud development. *Nature* 422 317–322. 10.1038/nature01458 12646922 PMC2424170

[B30] Kahler R. A. Westendorf J. J. (2003). Lymphoid enhancer factor-1 and beta-catenin inhibit Runx2-dependent transcriptional activation of the osteocalcin promoter. *J. Biol. Chem.* 278 11937–11944. 10.1074/jbc.M211443200 12551949

[B31] Karner C. M. Das A. Ma Z. Self M. Chen C. Lum L. (2011). Canonical Wnt9b signaling balances progenitor cell expansion and differentiation during kidney development. *Development* 138 1247–1257. 10.1242/dev.057646 21350016 PMC3050658

[B32] Kispert A. Vainio S. Mcmahon A. P. (1998). Wnt-4 is a mesenchymal signal for epithelial transformation of metanephric mesenchyme in the developing kidney. *Development* 125 4225–4234. 9753677 10.1242/dev.125.21.4225

[B33] Kispert A. Vainio S. Shen L. Rowitch D. H. Mcmahon A. P. (1996). Proteoglycans are required for maintenance of Wnt-11 expression in the ureter tips. *Development* 122 3627–3637. 8951078 10.1242/dev.122.11.3627

[B34] Kovacs G. Billfeldt N. K. Farkas N. Dergez T. Javorhazy A. Banyai D. (2017). Cytoplasmic expression of beta-catenin is an independent predictor of progression of conventional renal cell carcinoma: a simple immunostaining score. *Histopathology* 70 273–280. 10.1111/his.13059 27501523

[B35] Lee L. M. Y. Leung C.-Y. Tang W. W. C. Choi H.-L. Leung Y.-C. Mccaffery P. J. (2012). A paradoxical teratogenic mechanism for retinoic acid. *Proc. Natl. Acad. Sci. U.S.A.* 109 13668–13673. 10.1073/pnas.1200872109 22869719 PMC3427051

[B36] Leow C. C. Romero M. S. Ross S. Polakis P. Gao W.-Q. (2004). Hath1, down-regulated in colon adenocarcinomas, inhibits proliferation and tumorigenesis of colon cancer cells. *Cancer Res.* 64 6050–6057. 10.1158/0008-5472.can-04-0290 15342386

[B37] Levinson R. S. Batourina E. Choi C. Vorontchikhina M. Kitajewski J. Mendelsohn C. L. (2005). Foxd1-dependent signals control cellularity in the renal capsule, a structure required for normal renal development. *Development* 132 529–539. 10.1242/dev.01604 15634693

[B38] Li C.-M. Guo M. Borczuk A. Powell C. A. Wei M. Thaker H. M. (2002). Gene expression in Wilms’ tumor mimics the earliest committed stage in the metanephric mesenchymal-epithelial transition. *Am. J. Pathol.* 160 2181–2190. 10.1016/s0002-9440(10)61166-2 12057921 PMC1850829

[B39] Li M. H. Yamase H. Ferrer F. (2010). Characterization of a WiT49 cell line derived orthotopic model of Wilms tumor. *Pediatr. Blood Cancer* 54 316–318. 10.1002/pbc.22205 19824073 PMC2834252

[B40] Li Y. Wang L. Ai W. He N. Zhang L. Du J. (2017). Regulation of retinoic acid synthetic enzymes by WT1 and HDAC inhibitors in 293 cells. *Int. J. Mol. Med.* 40 661–672. 10.3892/ijmm.2017.3051 28677722 PMC5547963

[B41] Lin Y. Liu A. Zhang S. Ruusunen T. Kreidberg J. A. Peltoketo H. (2001). Induction of ureter branching as a response to Wnt-2b signaling during early kidney organogenesis. *Dev. Dyn.* 222 26–39. 10.1002/dvdy.1164 11507767

[B42] Lochhead P. A. Coghlan M. Rice S. Q. J. Sutherland C. (2001). Inhibition of GSK-3 selectively reduces glucose-6-phosphatase and phosphoenolpyruvate carboxykinase gene expression. *Diabetes* 50 937–946. 10.2337/diabetes.50.5.937 11334436

[B43] Ma J. (2005). Crossing the line between activation and repression. *Trends Genet.* 21 54–59. 10.1016/j.tig.2004.11.004 15680515

[B44] Magella B. Adam M. Potter A. S. Venkatasubramanian M. Chetal K. Hay S. B. (2018). Cross-platform single cell analysis of kidney development shows stromal cells express Gdnf. *Dev. Biol.* 434 36–47. 10.1016/j.ydbio.2017.11.006 29183737 PMC5930237

[B45] Marlier A. Gilbert T. (2004). Expression of retinoic acid-synthesizing and -metabolizing enzymes during nephrogenesis in the rat. *Gene Expr. Patterns* 5 179–185. 10.1016/j.modgep.2004.08.005 15567713

[B46] Marose T. D. Merkel C. E. Mcmahon A. P. Carroll T. J. (2008). β-Catenin is necessary to keep cells of ureteric bud/Wolffian duct epithelium in a precursor state. *Dev. Biol.* 314 112–126. 10.1016/j.ydbio.2007.11.016 18177851 PMC2699621

[B47] Masiá S. Alvarez S. De Lera A. R. Barettino D. (2007). Rapid, nongenomic actions of retinoic acid on phosphatidylinositol-3-kinase signaling pathway mediated by the retinoic acid receptor. *Mol. Endocrinol.* 21 2391–2402. 10.1210/me.2007-0062 17595318

[B48] Matt N. Dupé V. Garnier J.-M. Dennefeld C. Chambon P. Mark M. (2005). Retinoic acid-dependent eye morphogenesis is orchestrated by neural crest cells. *Development* 132 4789–4800. 10.1242/dev.02031 16207763

[B49] Mendelsohn C. Batourina E. Fung S. Gilbert T. Dodd J. (1999). Stromal cells mediate retinoid-dependent functions essential for renal development. *Development* 126 1139–1148. 10021334 10.1242/dev.126.6.1139

[B50] Mengelbier L. H. Bexell D. Sehic D. Ciornei C. D. Gisselsson D. (2014). Orthotopic Wilms tumor xenografts derived from cell lines reflect limited aspects of tumor morphology and clinical characteristics. *Pediatr. Blood Cancer* 61 1949–1954. 10.1002/pbc.25131 25044705

[B51] Mohri Y. Oyama K. Akamatsu A. Kato S. Nishimori K. (2011). Lgr4-deficient mice showed premature differentiation of ureteric bud with reduced expression of Wnt effector Lef1 and Gata3. *Dev. Dyn.* 240 1626–1634. 10.1002/dvdy.22651 21523854

[B52] Napoli J. L. (2012). Physiological insights into all-trans-retinoic acid biosynthesis. *Biochim. Biophys. Acta* 1821 152–167. 10.1016/j.bbalip.2011.05.004 21621639 PMC3179567

[B53] Napoli J. L. (2017). Cellular retinoid binding-proteins, CRBP, CRABP, FABP5: effects on retinoid metabolism, function and related diseases. *Pharmacol. Ther.* 173 19–33. 10.1016/j.pharmthera.2017.01.004 28132904 PMC5408321

[B54] Naylor R. W. Skvarca L. B. Thisse C. Thisse B. Hukriede N. A. Davidson A. J. (2016). BMP and retinoic acid regulate anterior–posterior patterning of the non-axial mesoderm across the dorsal–ventral axis. *Nat. Commun.* 7:12197. 10.1038/ncomms12197 27406002 PMC4947171

[B55] Niederreither K. Fraulob V. Garnier J.-M. Chambon P. Dollé P. (2002). Differential expression of retinoic acid-synthesizing (RALDH) enzymes during fetal development and organ differentiation in the mouse. *Mech. Dev.* 110 165–171. 10.1016/s0925-4773(01)00561-5 11744377

[B56] Nusse R. Clevers H. (2017). Wnt/β-catenin signaling, disease, and emerging therapeutic modalities. *Cell* 169 985–999. 10.1016/j.cell.2017.05.016 28575679

[B57] Pan X. Karner C. M. Carroll T. J. (2017). Myc cooperates with β-catenin to drive gene expression in nephron progenitor cells. *Development* 144 4173–4182. 10.1242/dev.153700 28993399 PMC5719246

[B58] Park J.-S. Ma W. O’Brien L. L. Chung E. Guo J.-J. Cheng J.-G. (2012). Six2 and Wnt regulate self-renewal and commitment of Nephron progenitors through shared gene regulatory networks. *Dev. Cell* 23 637–651. 10.1016/j.devcel.2012.07.008 22902740 PMC3892952

[B59] Park J.-S. Valerius M. T. Mcmahon A. P. (2007). Wnt/β-catenin signaling regulates nephron induction during mouse kidney development. *Development* 134 2533–2539. 10.1242/dev.006155 17537789

[B60] Piersma A. H. Hessel E. V. Staal Y. C. (2017). Retinoic acid in developmental toxicology: teratogen, morphogen and biomarker. *Reprod. Toxicol.* 72 53–61. 10.1016/j.reprotox.2017.05.014 28591664

[B61] Piskunov A. Rochette-Egly C. (2011). A retinoic acid receptor RARα pool present in membrane lipid rafts forms complexes with G protein αQ to activate p38MAPK. *Oncogene* 31 3333–3345. 10.1038/onc.2011.499 22056876

[B62] Przepiorski A. Sander V. Tran T. Hollywood J. A. Sorrenson B. Shih J. H. (2018). A simple bioreactor-based method to generate kidney organoids from pluripotent stem cells. *Stem Cell Rep.* 11 470–484. 10.1016/j.stemcr.2018.06.018 30033089 PMC6092837

[B63] Rao S. S. P. Huntley M. H. Durand N. C. Stamenova E. K. Bochkov I. D. Robinson J. T. (2014). A 3D map of the human genome at kilobase resolution reveals principles of chromatin looping. *Cell* 159 1665–1680. 10.1016/j.cell.2014.11.021 25497547 PMC5635824

[B64] Rivera M. N. Haber D. A. (2005). Wilms’ tumour: connecting tumorigenesis and organ development in the kidney. *Nat. Rev. Cancer* 5 699–712. 10.1038/nrc1696 16110318

[B65] Rosselot C. Spraggon L. Chia I. Batourina E. Riccio P. Lu B. (2010). Non-cell-autonomous retinoid signaling is crucial for renal development. *Development* 137 283–292. 10.1242/dev.040287 20040494 PMC2799161

[B66] Sima A. Parisotto M. Mader S. Bhat P. V. (2009). Kinetic characterization of recombinant mouse retinal dehydrogenase types 3 and 4 for retinal substrates. *Biochim. Biophys. Acta* 1790 1660–1664. 10.1016/j.bbagen.2009.09.004 19766701

[B67] Smartt H. J. M. Greenhough A. Ordóñez-Morán P. Talero E. Cherry C. A. Wallam C. A. (2012). β-catenin represses expression of the tumour suppressor 15-prostaglandin dehydrogenase in the normal intestinal epithelium and colorectal tumour cells. *Gut* 61 1306–1314. 10.1136/gutjnl-2011-300817 22082586

[B68] Spencer G. J. Utting J. C. Etheridge S. L. Arnett T. R. Genever P. G. (2006). Wnt signalling in osteoblasts regulates expression of the receptor activator of NFκB ligand and inhibits osteoclastogenesis in vitro. *J. Cell Sci.* 119 1283–1296. 10.1242/jcs.02883 16522681

[B69] Stark K. Vainio S. Vassileva G. Mcmahon A. P. (1994). Epithelial transformation of metanephric mesenchyme in the developing kidney regulated by Wnt-4. *Nature* 372 679–683. 10.1038/372679a0 7990960

[B70] Suryawanshi A. Manicassamy S. (2015). Tumors induce immune tolerance through activation of beta-catenin/TCF4 signaling in dendritic cells: a novel therapeutic target for cancer immunotherapy. *Oncoimmunology* 4:e1052932. 10.1080/2162402X.2015.1052932 26587326 PMC4635893

[B71] Taguchi A. Nishinakamura R. (2017). Higher-order kidney organogenesis from pluripotent stem cells. *Cell Stem Cell* 21 730–746.e6. 29129523 10.1016/j.stem.2017.10.011

[B72] Takasato M. Er P. X. Chiu H. S. Maier B. Baillie G. J. Ferguson C. (2016). Kidney organoids from human iPS cells contain multiple lineages and model human nephrogenesis. *Nature* 536:238. 10.1038/nature17982 27120161

[B73] Tanigawa S. Wang H. Yang Y. Sharma N. Tarasova N. Ajima R. (2011). Wnt4 induces nephronic tubules in metanephric mesenchyme by a non-canonical mechanism. *Dev. Biol.* 352 58–69. 10.1016/j.ydbio.2011.01.012 21256838 PMC3049843

[B74] Terada N. Karim M. R. Izawa T. Kuwamura M. Yamate J. (2017). Immunolocalization of beta-catenin, E-cadherin and N-cadherin in neonate and adult rat kidney. *J. Vet. Med. Sci.* 79 1785–1790. 10.1292/jvms.17-0439 28993569 PMC5709553

[B75] Veeman M. T. Slusarski D. C. Kaykas A. Louie S. H. Moon R. T. (2003). Zebrafish prickle, a modulator of noncanonical Wnt/Fz signaling, regulates gastrulation movements. *Curr. Biol.* 13 680–685. 10.1016/s0960-9822(03)00240-9 12699626

[B76] Wang P. Chen Y. Yong J. Cui Y. Wang R. Wen L. (2018). Dissecting the global dynamic molecular profiles of human fetal kidney development by single-cell RNA sequencing. *Cell Rep.* 24 3554–3567.e3. 10.1016/j.celrep.2018.08.056 30257215

[B77] Wang X. Penzes P. Napoli J. L. (1996). Cloning of a cDNA encoding an aldehyde dehydrogenase and its expression in Escherichia coli, Recognition of retinal as substrate. *J. Biol. Chem.* 271 16288–16293. 10.1074/jbc.271.27.16288 8663198

[B78] Wehner D. Cizelsky W. Vasudevaro M. D. Ozhan G. Haase C. Kagermeier-Schenk B. (2014). Wnt/beta-catenin signaling defines organizing centers that orchestrate growth and differentiation of the regenerating zebrafish caudal fin. *Cell Rep.* 6 467–481. 10.1016/j.celrep.2013.12.036 24485658

[B79] Wingert R. A. Selleck R. Yu J. Song H. D. Chen Z. Song A. (2007). The cdx genes and retinoic acid control the positioning and segmentation of the Zebrafish pronephros. *PLoS Genet.* 3:e189. 10.1371/journal.pgen.0030189 17953490 PMC2042002

[B80] Yu H. Ye X. Guo N. Nathans J. (2012). Frizzled 2 and frizzled 7 function redundantly in convergent extension and closure of the ventricular septum and palate: evidence for a network of interacting genes. *Development* 139 4383–4394. 10.1242/dev.083352 23095888 PMC3509732

[B81] Yu J. Carroll T. J. Rajagopal J. Kobayashi A. Ren Q. Mcmahon A. P. (2008). A Wnt7b-dependent pathway regulates the orientation of epithelial cell division and establishes the cortico-medullary axis of the mammalian kidney. *Development* 136 161–171. 10.1242/dev.022087 19060336 PMC2685965

[B82] Zhao D. Mccaffery P. Ivins K. J. Neve R. L. Hogan P. Chin W. W. (1996). Molecular identification of a major retinoic-acid-synthesizing enzyme, a retinaldehyde-specific dehydrogenase. *Eur. J. Biochem.* 240 15–22. 10.1111/j.1432-1033.1996.0015h.x 8797830

