# Supplementary figures and images for: Inhibition of GSK3 Represses the Expression of Retinoic Acid Synthetic Enzyme ALDH1A2 via Wnt/β-Catenin Signaling in WiT49 Cells
Source: Front Cell Dev Biol. 2020 Mar 17;8:94. doi: 10.3389/fcell.2020.00094 (PMC7092725; doi:10.3389/fcell.2020.00094)

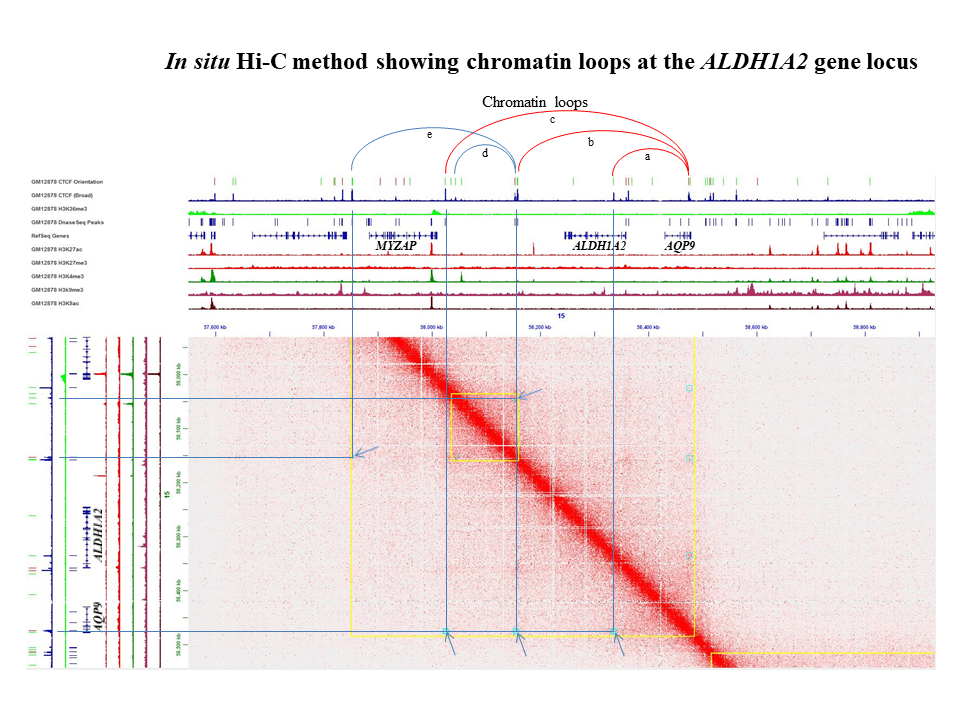

Supplement: FIGURE S1 — CCCTC-binding factor (CTCF) binding to ALDH1A2 gene locus and formation of DNA loops in GM12878 cells. The arrows show the two distal DNA sites where the chromatin loops bind to each other (the straight line extending from the point directs to two DNA sites; in most cases, the CTCF binding site). The red (a, b, c) and blue (d, e) arcs indicate the extent of each chromatin loop, and the red chromatin loop (a, b, c) totally or partially covers the ALDH1A2 gene. This figure was based on a previous report using the Hi-C method (Rao et al., 2014). The report detailed a Hi-C experiment conducted to examine the genome-wide chromatin loop in the GM12878 cell line. The data were integrated with the ChIP-seq data of CTCF, H3K36me3 (active mark), H3K27ac (active mark), H3K27me3 (repressive mark), H3K4me3 (active mark), H3K9me3 (repressive mark), H3K9ac (active mark), and Dnase Seq data. Since the chromatin loop is relatively stable among species and different tissues, the data of chromatin loop at ALDH1A2 locus obtained from the lymphoblastoid cell line GM12878 give us clues on how the chromatin at ALDH1A2 gene locus is organized. The data show that ALDH1A2 promoter, intron1G, and the BSCR element are likely to be within the same chromatin loop. Thus, they may have close spatial proximity and less physical segmentation, suggesting that they have the potential to interact with each other and may be relevant to β-catenin recruitment and regulation of ALDH1A2 expression. [file Image_1.TIF]

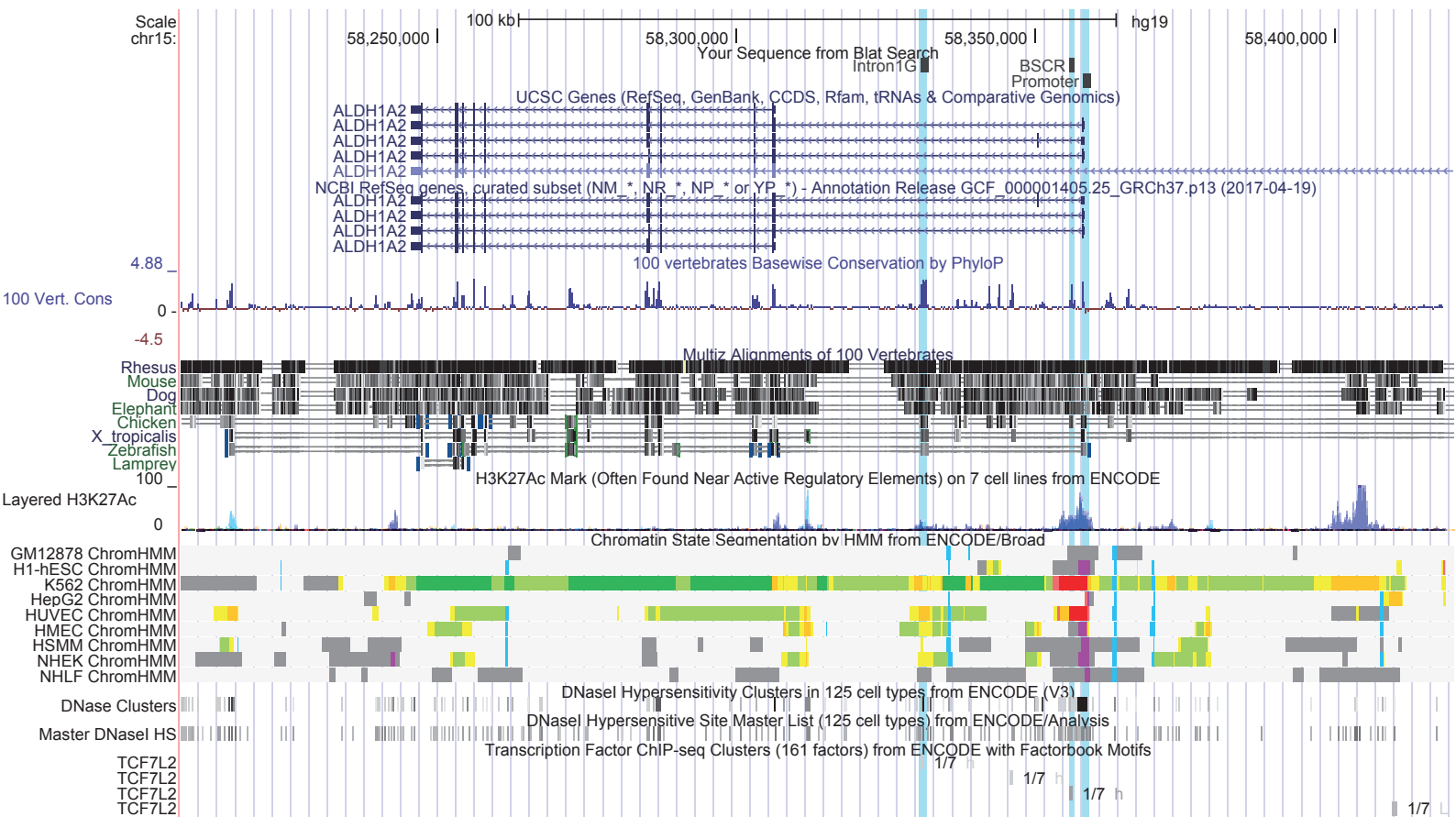

Supplement: FIGURE S2 — The chromatin state of the ALDH1A2 gene. Intron1G, BSCR, and ALDH1A2 promoter sequences were searched on genome on the UCSC browser and highlighted in a light blue color. The lane “100 vertebrates Basewise Conservation by PhyloP” shows that the ALDH1A2 promoter, intron1G, and BSCR element are all evolutionally conserved. The lane “Chromatin State Segmentation by HMM from ENCODE/Broad” shows that ALDH1A2 promoter and intron1G are in an open chromatin state in nine cell lines, namely GM12878, H1-hESC, K562, HepG2, HUVEC, HMEC, HSMM, NHEK, and NHLF. The lane “DNaseI Hypersensitivity Clusters in 125 cell types from ENCODE (V3)” also shows that the ALDH1A2 promoter and intron1G are in an open chromatin state. The lane “Transcription Factor ChIP-seq Clusters (161 factors) from ENCODE with Factorbook Motifs” shows moderate TCF7L2 binding on the intron1G and BSCR site. [file Image_2.PDF]
